# Cripto is essential to capture mouse epiblast stem cell and human embryonic stem cell pluripotency

**DOI:** 10.1038/ncomms12589

**Published:** 2016-09-02

**Authors:** Alessandro Fiorenzano, Emilia Pascale, Cristina D'Aniello, Dario Acampora, Cecilia Bassalert, Francesco Russo, Gennaro Andolfi, Mauro Biffoni, Federica Francescangeli, Ann Zeuner, Claudia Angelini, Claire Chazaud, Eduardo J. Patriarca, Annalisa Fico, Gabriella Minchiotti

**Affiliations:** 1 Stem Cell Fate Laboratory, Institute of Genetics and Biophysics ‘A. Buzzati-Traverso', CNR, Via Pietro Castellino 111, 80131 Naples, Italy; 2 Institute of Genetics and Biophysics ‘A. Buzzati-Traverso', CNR, 80131 Naples, Italy; 3 IRCCS Neuromed, 860077 Pozzilli (IS), Italy; 4 Inserm, UMR1103, F-63001; CNRS, UMR6293, F-63001; Université Clermont Auvergne, Laboratoire GReD, BP 10448, Clermont-Ferrand F-63000, France; 5 Institute for Applied Mathematics ‘Mauro Picone', CNR, 80131, Naples, Italy; 6 Department of Hematology, Oncology and Molecular Medicine, Istituto Superiore di Sanità, Rome 00161, Italy

## Abstract

Known molecular determinants of developmental plasticity are mainly transcription factors, while the extrinsic regulation of this process has been largely unexplored. Here we identify Cripto as one of the earliest epiblast markers and a key extracellular determinant of the naive and primed pluripotent states. We demonstrate that Cripto sustains mouse embryonic stem cell (ESC) self-renewal by modulating Wnt/β-catenin, whereas it maintains mouse epiblast stem cell (EpiSC) and human ESC pluripotency through Nodal/Smad2. Moreover, we provide unprecedented evidence that Cripto controls the metabolic reprogramming in ESCs to EpiSC transition. Remarkably, Cripto deficiency attenuates ESC lineage restriction *in vitro* and *in vivo*, and permits ESC transdifferentiation into trophectoderm lineage, suggesting that Cripto has earlier functions than previously recognized. All together, our studies provide novel insights into the current model of mammalian pluripotency and contribute to the understanding of the extrinsic regulation of the first cell lineage decision in the embryo.

## Abstract (web summary)

Stem cell plasticity is crucial for early embryo development and the differentiation of stem cells. Here, the authors show that the extracellular protein Cripto sustains mouse ESC self-renewal and maintains mouse EpiSC as well as human ESC pluripotency and controls the metabolic reprogramming in ESCs to EpiSC transition.

## Graphical abstract

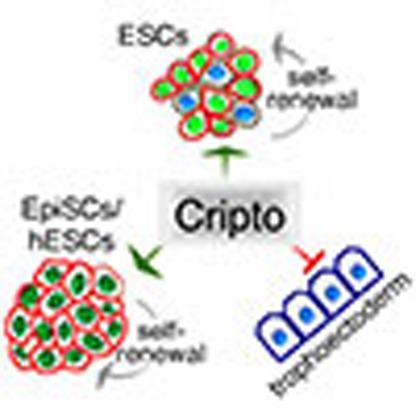

In mammals, pluripotency is maintained in the inner cell mass (ICM) of early embryo during the formation of epiblast (EPI), which is shielded from extraembryonic differentiation and concomitantly gains the capacity to generate all cell types of the organism. The formation of these lineages results from two subsequent cell fate decisions. The first, occurring at the 16- to 32-cell stage, determines the specification of trophectoderm (TE) and ICM; the second cell-fate decision controls the formation of the primitive endoderm (PrE) and EPI within the ICM. The emergence of the pluripotent EPI and PrE lineages within the ICM involves initial co-expression of lineage-associated markers followed by a salt-and-pepper distribution of lineage-biased cells until the time of implantation when cell lineages determination is established^1^,^2^. Two distinct states of mouse pluripotency can be captured *in vitr*o, that is, embryonic stem cells (ESCs) and EPI stem cells (EpiSCs), which reflect the ground-state naive and the primed EPI, respectively^3^. Although ESCs grow as packed domed colonies and are stabilized by leukemia inhibitory factor (LIF)/Stat3 signalling, mEpiSCs depend on basic fibroblast growth factor (bFGF) and transforming growth factor-β (TGFβ)/Activin signalling and are characterized by a flattened morphology^4^,^5^. Mouse EpiSCs can be also generated *in vitro* from ESCs, providing a useful model system to study pluripotent state transition that occurs at implantation^6^. Unlike mouse ESCs, human ESCs (hESCs) depend on TGFβ/Activin signalling and share common features of mEpiSCs with respect to growth requirements, morphology, clonogenicity and gene expression patterns^3^. Mouse ESC (mESC) cultures are not homogeneous but comprise dynamically interchanging subpopulations^7^,^8^. This heterogeneity probably reflects the developmental plasticity of the early mouse embryo; however, a mechanistic understanding of this metastability *in vitro* is still far from complete. Specifically, which is the precise correlation of these different pluripotency states with the *in vivo* equivalents is still a question of debate. Known molecular markers of such plasticity are mainly transcription factors operating within a pluripotency gene regulatory network^9^. More recently, metabolites are emerging as key regulators of stem cell plasticity, acting as epigenetic modifiers^10^,^11^; however, much less is known on the role of microenvironment. Indeed, elucidation of the extrinsic mechanisms that control stem cell plasticity is crucial for understanding both early embryo development and controlling the differentiation potential of pluripotent stem cells^12^. In the attempt to shed lights on this issue, we focused on the glycosylphosphatidylinositol (GPI)-anchored extracellular protein Cripto. Cripto is a key developmental factor and a multifunctional signalling molecule^13^. In the mouse embryo, *Cripto* is essential for primitive streak formation and patterning of the anterior–posterior axis during gastrulation^14^ and it negatively regulates ESC neural differentiation while permitting cardiac differentiation^15^. Although largely considered as a stem cell surface marker^16^, no studies so far have directly investigated its functional role in pluripotency. In this study, we report the consequences of genetic and pharmacological modulation of Cripto signalling on the generation and/or maintenance of mEpiSCs and hESCs.

## Results

### Cripto heterogeneity in the early blastocyst and ESCs

**Figure 1:**
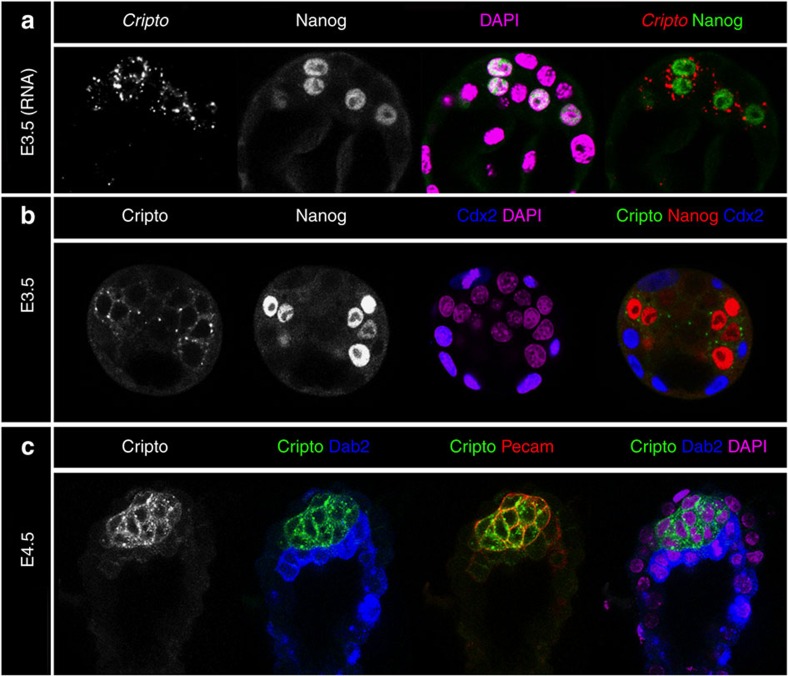
Cripto is specifically expressed in EPI cells. (**a**) FISH and (**b**) immunofluorescence analyses of Cripto expression at E3.5. Both *Cripto* RNA and protein are present in Nanog-expressing cells. (**c**) By E4.5, *Cripto* remains expressed in the EPI, labelled by Pecam1 and is absent from the PrE revealed by Disabled 2 (Dab2) and the TE.

In the pre-implantation embryo (E3.5), Cripto messenger RNA and protein were present in the blastomeres of the ICM in a salt-and-pepper pattern (Fig. 1). Indeed, Cripto expression was highly enriched in Nanog-expressing cells, whereas it was absent in PrE cells and TE marked by *Cdx2* (Fig. 1a,b)^17^. After cell sorting at E4.5, Cripto was co-expressed with Pecam1, a membrane EPI marker, but not Disabled 2, which labels the PrE (Fig. 1c), as was previously shown^18^,^19^. Thus, *in situ* expression analysis revealed that Cripto is homogeneously expressed in EPI cells only as early as EPI versus PrE specification occurs within the ICM, earlier than previously reported^18^,^19^. Cripto remains strongly expressed in the maturing EPI until gastrulation where it becomes restricted to the primitive streak^14^,^20^.

**Figure 2:**
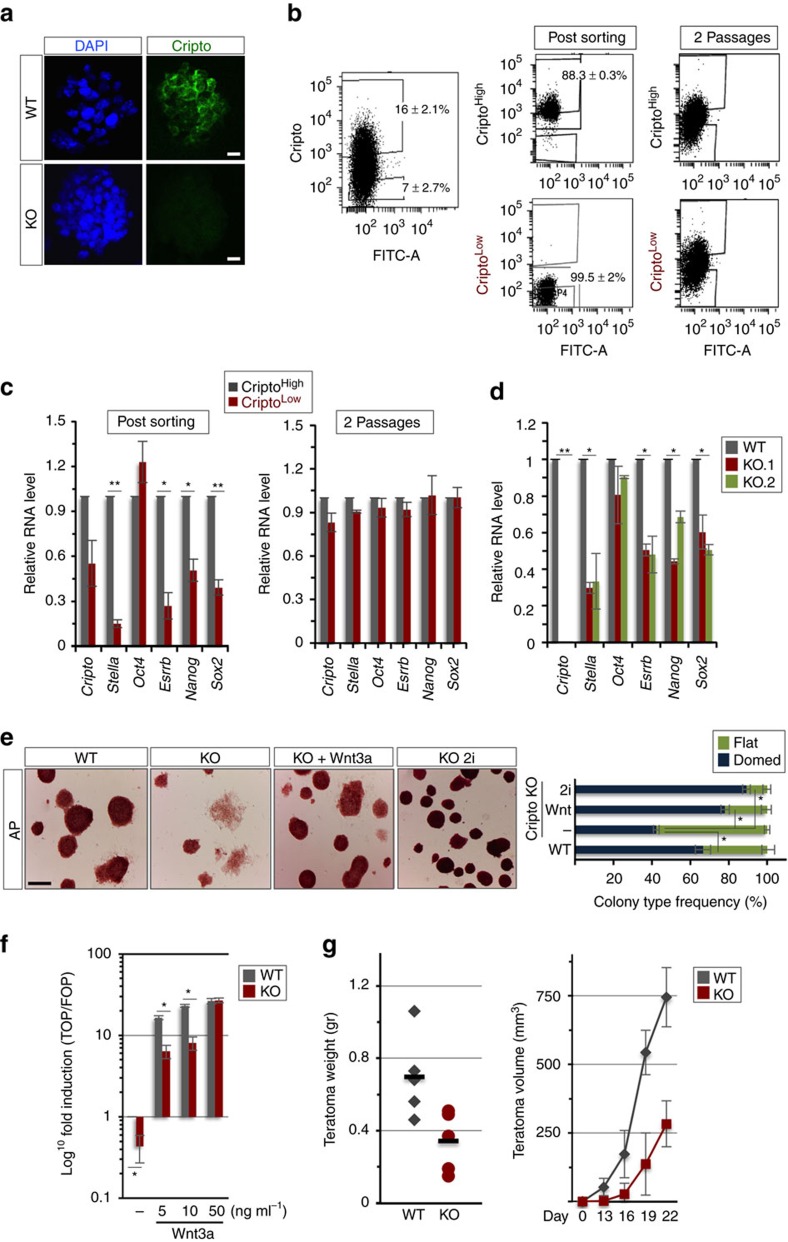
Cripto sustains mESC self-renewal by modulating canonical Wnt signalling. (**a**) Immunofluorescence of Cripto on non-permeabilized WT and Cripto KO ESCs. Nuclei were stained with 4,6-diamidino-2-phenylindole (DAPI). Scale bar, 75 μm. (**b**) FACS-based separation of Cripto^High^ and Cripto^Low^ in serum/LIF mESCs. Data are mean±s.e.m. (*n*= 3). (**c**) qPCR of Cripto and pluripotency genes' expression on Cripto^High^ and Cripto^Low^ cells immediately after sorting and after two passages in culture. Relative RNA level was normalized to *Gapdh*; data are mean±s.e.m. (*n*=3; *P<0.01 and **P<0.005). (**d**) qPCR of pluripotency genes' expression in WT and two independent Cripto KO ESC clones (KO.1 and KO.2) in serum/LIF culture conditions. Relative RNA level was normalized to *Gapdh*; data are mean±s.e.m. (*n*=3; *P<0.01 and **P<0.005). (**e**) Representative pictures (scale bar, 100 μm) of AP- stained and colony-type frequency (domed versus flat) of colonies generated from WT, Cripto KO, KO+Wnt3a (50 ng ml^−1^) and KO 2i ESCs, at day 6 after plating (∼100 colonies scored per condition). (**f**) TOP flash reporter activation in WT and Cripto KO ESCs in baseline conditions and following Wnt3a stimulation at indicated concentrations. Data are mean±s.e.m. (*n*=3); *P<0.01. (**g**) Effect of Cripto depletion on teratomas formation. Quantitative analysis of tissue weights (left panel) and temporal kinetic analysis (right panel) of teratomas growth (volume, mm^3^). Data are mean±s.e.m. (five mice per group).

To assess whether the heterogeneous distribution of Cripto *in vivo* was retained *in vitro*, we analysed Cripto protein distribution in serum/LIF ESC cultures^9^. Immunofluorescence and fluorescence-activated cell sorting (FACS) analysis showed a heterogeneous pattern of surface Cripto protein expression (Fig. 2a,b) and revealed that ESCs clearly segregated in two cell populations of Cripto^High^ and Cripto^Low^, which rapidly returned to the equilibrium under ESC self-renewing conditions (Fig. 2b). Interestingly, Cripto expression increased in 2i/LIF culture conditions (Supplementary Fig. 1a), in which ESCs show lower expression of lineage-associated genes and are closer to the pluripotent cells of the ICM^6^,^21^. The dynamic equilibrium of distinct functional states in serum ESCs is characterized by the heterogeneous expression of different pluripotency transcription factors, for example, *Nanog*, *Stella (Dppa3)* and *Esrrb*. We thus evaluated the expression of pluripotency markers in the Cripto^Low^ and Cripto^High^ cell populations and found that it was significantly reduced in Cripto^Low^ post sorting, whereas it rapidly returned to the equilibrium after *in vitro* culture (Fig. 2c). On the contrary, *Oct4*, which is homogeneously expressed in serum/LIF ESCs^22^, was expressed at comparable levels in the two subpopulations (Fig. 2c). These data indicated that Cripto protein levels dynamically fluctuate in ESCs and correlate with the metastable expression of key pluripotency transcription factors. A similar heterogeneous and fluctuating expression of Cripto has been recently described in patient-derived colon cancer stem cells, where Cripto positivity oscillates in correspondence to increased stemness states^23^, and in a human glioblastoma cell line^24^. Interestingly, fluctuations of Cripto protein levels occurred on small variations of the transcript (Fig. 2c), suggesting that regulation does not primarily occur at a transcriptional level as described in colon cancer stem cells^23^.

### Decreased self-renewal properties of Cripto KO ESCs

To assess the relevance of the correlation between *Cripto* and the expression of pluripotency genes to potency and fate choice, we analysed two independent Cripto Knock Out (KO) ESC (KO.1 and KO.2) clones. Similar to that observed in Cripto^Low^ and Cripto^High^ cell populations, the pluripotency genes were downregulated in both Cripto KO ESC clones compared with Control (Fig. 2d). Despite this molecular signature, Cripto KO ESCs propagated at high density retained the capacity to form tightly packed dome*-*shaped colonies. Nevertheless, when Cripto KO ESCs were plated at low density in a colony-formation assay, the colonies with a flat morphology and a partial/low alkaline phosphate (AP) staining significantly increased, at the expense of the typical dome-like AP^+^ ESC colonies (Fig. 2e). Interestingly, this phenotype was rescued by the addition of either recombinant Wnt3a (Fig. 2e), which is a key regulator of ESC self-renewal^25^, or the glycogen synthase kinase 3 inhibitor CHIR99021, a selective inhibitor of β-catenin degradation, as well as in 2i/LIF culture conditions (Fig. 2e and Supplementary Fig. 1b). We thus reasoned that Cripto deficiency might affect sensitivity to Wnt signalling and eventually ESC self-renewal *in vitro*. We thus measured Wnt/β-catenin activity in wild-type (WT) and Cripto KO ESCs using the TOP flash/luciferase reporter construct. Cripto KO ESCs showed reduced luciferase activity already at baseline conditions, which persisted on stimulation with increasing doses (5–10 ng ml^−1^) of Wnt3a. On the contrary, addition of high doses of Wnt3a (50 ng ml^−1^) to WT and Cripto KO ESCs resulted in similar activation of the reporter (Fig. 2f). In line with these findings, Cripto KO ESCs showed decreased levels of nuclear β-catenin, which increased on chemical activation of Wnt/β-catenin pathway (Supplementary Fig. 1c). Accordingly, expression of the Wnt target gene *Lef1* was downregulated in two independent Cripto KO ESC clones (Supplementary Fig. 1d). Interestingly, Cripto is able to positively modulate Wnt signalling in human mammary epithelial and mouse teratocarcinoma cells, but only on Wnt administration^26^. Maintenance of ESCs *in vitro* also depends on extracellular signalling by LIF and Bmp4. Stimulation of WT and Cripto KO ESCs with either LIF or Bmp4 resulted in similar increase of the phosphorylation of the intracellular effectors Stat3 and Smad1/5, respectively (Supplementary Fig. 1e). All together, these findings indicate that Cripto genetic ablation reduced ESC self-renewal efficiency in fetal bovine serum (FBS)/LIF but not in 2i/LIF culture conditions^27^ and suggest that Cripto KO specifically altered Wnt response in ESCs. In line with the idea that *Cripto* KO reduced ESC self-renewal properties, we found substantial differences in the efficiency and latency of Cripto KO ESC-derived teratomas (Fig. 2g) but not in their histological composition (Supplementary Fig. 1f) as previously reported^28^.

### Cripto controls the metabolic switch in ESC→EpiSC transition

**Figure 3:**
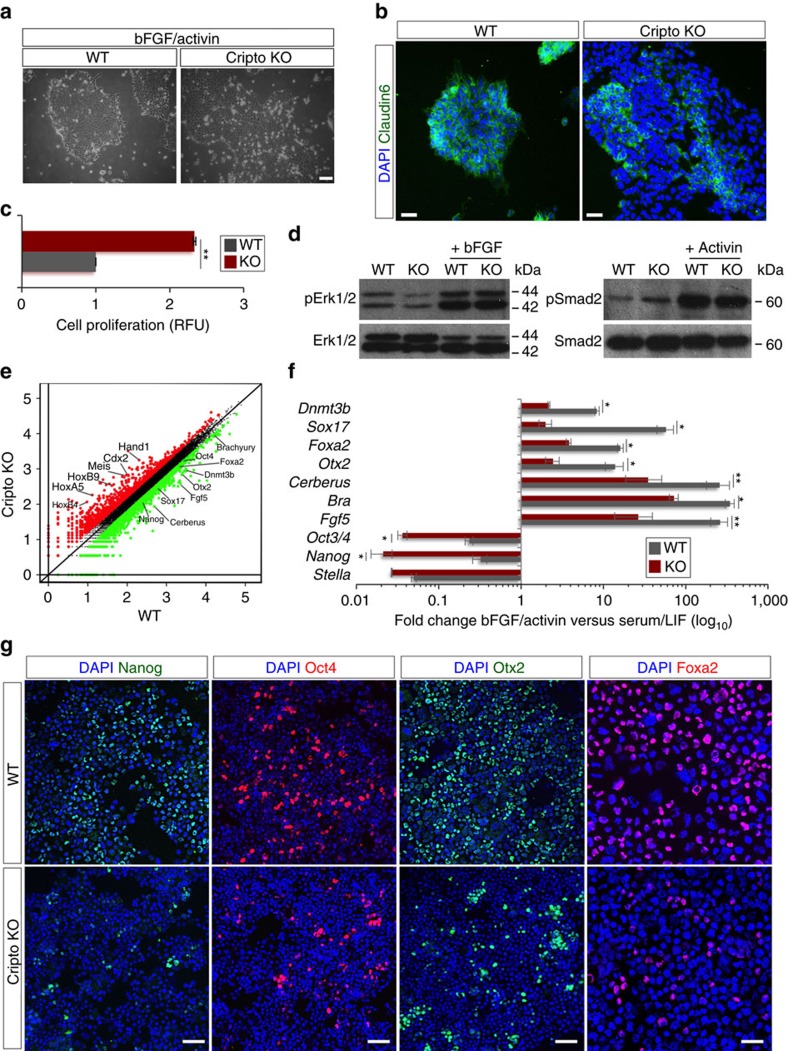
Cripto genetic ablation prevents ESC→EpiSC transition. (**a**) Representative photomicrographs of FGF/Activin (F/A) WT and Cripto KO cell colonies (scale bar, 100 μm). (**b**) Immunofluorescence of Claudin6 in F/A WT and Cripto KO cell colonies (scale bar, 75 μm). (**c**) Proliferation of F/A WT and Cripto KO cells measured by the CyQuant assay and expressed as relative fluorescence units (RFU). Data are mean±s.e.m. (*n*=3; **P<0.005). (**d**) Western blot analysis of WT and Cripto KO ESCs' response to bFGF and Activin, using pErk1/2, Erk1/2, pSmad2 and Smad2 antibodies. (**e**) Scatter plot of RNA-seq data shows differentially expressed genes in F/A Cripto KO versus WT cells. The black circles delineate the boundaries of <2-fold difference in gene expression levels. Genes showing a higher or lower expression level (log_10_ FC>0.2) in Cripto KO versus WT are indicated as red and green circles, respectively. (**f**) Effect of F/A on the expression of selected markers in WT and Cripto KO ESCs. Data are expressed as fold change of EpiSCs (F/A) versus ESCs (serum/LIF) after normalization to *Gapdh* and are mean±s.e.m. (*n*=3; *P<0.01 and **P<0.005). (**g**) Representative pictures of Nanog, Oct4, Otx2 (scale bar, 100 μm) and Foxa2 (scale bar, 75 μm) immunostaining in cytospinned F/A WT and Cripto KO cells. Nuclei were stained with 4,6-diamidino-2-phenylindole (DAPI).

**Figure 4:**
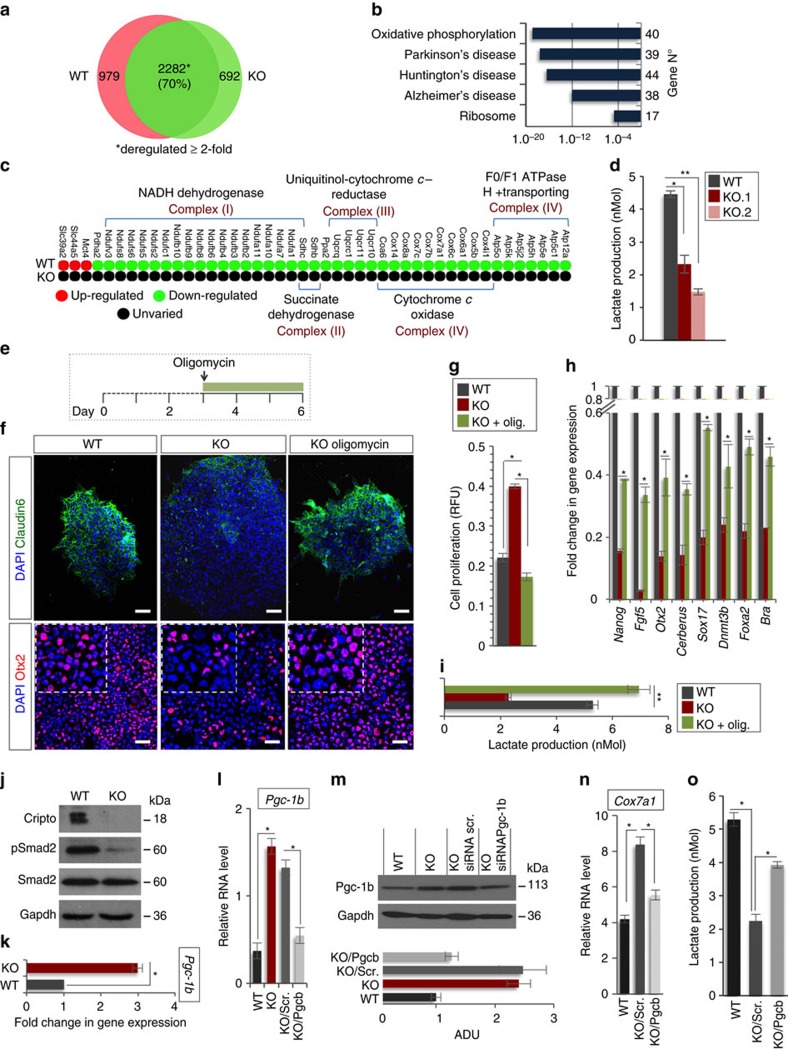
Cripto controls the metabolic reprogramming in ESC→EpiSC transition. (**a**) Venn diagram of genes differentially expressed (≥2-fold) or uniquely deregulated in Cripto KO versus WT ESC→EpiSC transition. (**b**) GO analysis (http://david.abcc.ncifcrf.gov) of protein coding genes (979) uniquely deregulated in WT ESC→EpiSC showing gene enrichment in KEGG Pathway. (**c**) Heatmap of genes involved in oxidative phosphorylation in WT and Cripto KO ESC→EpiSC transition. (**d**) Lactate concentration of F/A WT and Cripto KO cells. Two independent Cripto KO ESCs (KO.1 and KO.2) were used. Data are mean±s.e.m. (*n*=3; *P<0.01 and **P<0.005). (**e**) Schematic representation of the experimental procedure. (**f**) Representative immunofluorescence of Claudin6 and Otx2 in F/A WT, Cripto KO and Cripto KO+oligomycin colonies and cytospinned cells (scale bar, 75 μm). Inserts are higher magnification images of selected areas. (**g**) Proliferation of F/A WT, Cripto KO and Cripto KO+oligomycin cells measured by the CyQuant assay and expressed as relative fluorescence units (RFU). Data are mean±s.e.m. (*n*=3; *P<0.01). (**h**) qPCR of selected markers in F/A WT, Cripto KO and Cripto KO+oligomycin. Relative RNA level was normalized to *Gapdh*; data are mean±s.e.m. (*n*=3; *P<0.01). (**i**) Lactate concentration of F/A WT, Cripto KO and Cripto KO+oligomycin cells. Data are mean±s.e.m. (*n*=3; **P<0.005). (**j**) Western blot analysis of Cripto and p-Smad2 in F/A WT and Cripto KO cells. Smad2 and Gapdh were loading controls. (**k**) qPCR of *Pgc-1b*. Data are fold induction of Cripto KO versus WT F/A after normalization to *Gapdh* and are mean±s.e.m. (*n*=3; *P<0.01). (**l**) qPCR of *Pgc-1b* on F/A WT and Cripto KO cells transfected with two independent Pgc-1b small interfering RNAs (siRNAs) (KO/siPgcb) or control siRNA (KO/Scr.) at day 3 during ESC→EpiSC. Data are fold induction of Cripto KO versus WT and KO/Scr. versus KO/Pgcb after normalization to *Gapdh* and are mean±s.e.m. (*n*=3; *P<0.01). (**m**) Western blot analysis of Pgc-1b in F/A WT, Cripto KO, Cripto KO/Scr. and Cripto KO/siPgc-1b (KO/Pgcb). Gapdh were loading controls. The densitometric analysis is expressed in arbitrary unit (ADU) as the Pgc-1b/Gadph ratio. Data are mean±s.e.m. (*n*=2). (**n**) qPCR of *Cox7a1* and (**o**) lactate concentration in culture medium of F/A WT, Cripto KO/Scr and Cripto KO/Pgcb. Relative RNA level was normalized to *Gapdh.* Data are mean±s.e.m. (*n*=3; *P<0.01).

Our *in vivo* and *in vitro* findings led us to hypothesize that Cripto may have a functional role in the narrow window in which the cells within the ICM are primed to become EpiSCs. To assess this issue directly, WT and Cripto KO ESCs were treated with bFGF (F) and Activin (A), previously shown to permit *in vitro* EpiSC derivation and maintenance^6^. Control cells developed EpiSCs-like flat-shaped colonies with tightly packed cells and highly positive for the tight-junction protein Claudin6 (ref. 29) (Fig. 3a,b). Conversely, Cripto KO ESCs developed less compacted and morphologically highly heterogeneous colonies showing large areas of Claudin6-negative cells and increased proliferation (Fig. 3a–c and Supplementary Fig. 2a). Interestingly, surface Cripto protein was expressed at higher levels in WT EpiSCs than in serum/LIF ESCs (Supplementary Fig. 2b) consistent with its *in vivo* expression, which is higher in late blastocyst (Fig. 1b,c). We first verified that Activin and/or FGF signalling were efficiently induced in Cripto KO ESCs (Fig. 3d and Supplementary Fig. 2c) and then performed RNA-sequencing (RNA-Seq) transcriptome profiling of WT and Cripto KO ESC→EpiSC transition. As expected, culture conditions of EpiSC induction (F/A) extensively modified the transcriptome of both WT and Cripto KO ESCs, deregulating ∼3,000 protein-coding genes (≥2-fold, posterior probability (PP)≥0.95; Supplementary Data 1,2). However, F/A-treated Cripto KO ESCs showed downregulation of both EpiSCs markers (*Fgf5*, *Otx2*, *Cerberus*, *Brachyury* (*Bra*), *Sox17* and *Foxa2*) and the pluripotency genes (*Oct4* and *Nanog*)^30^ (Fig. 3e). These results were further validated by quantitative PCR (qPCR) analysis using two independent Cripto KO ESC clones, showing that this set of genes were significantly downregulated in both biological replicates (Fig. 3f and Supplementary Fig. 2d). Consistent with these findings, *Cripto* complementary DNA (cDNA) overexpression was able to fully rescue EpiSCs markers' expression in Cripto KO ESC→EpiSC transition (Supplementary Fig. 2e,f). Remarkably, immunofluorescence analysis revealed that the number of Nanog-, Oct4-, Otx2-, Foxa2- and Sox17-positive cells were all severely reduced in F/A Cripto KO cells (Fig. 3g and Supplementary Fig. 2g), thus further supporting the idea that ESC→EpiSC transition was impaired. Interestingly, besides a set of ∼2,300 (∼70%) common genes deregulated in both WT and Cripto KO ESC→EpiSC transition, we identified two different sets of genes that were uniquely deregulated (Fig. 4a and Supplementary Data 3). Specifically, Gene Ontology (GO) analysis showed that a large cluster of genes coding for components of the five respiratory electron transport complexes, which are involved in mitochondrial oxidative phosphorylation, were downregulated only in WT ESC→EpiSC transition (*P*-value=1.2 × 10^–19^), whereas their expression was unvaried in Cripto KO (Fig. 4b,c). Interestingly, one of the earliest events in the ESC→EpiSC transition is a dramatic metabolic switch, which converts a bivalent ESC metabolism to an exclusively glycolytic EpiSC metabolism^31^. To assess whether this metabolic reprogramming was affected in Cripto KO ESC→EpiSC transition, we measured lactate production as an indicator of the glycolytic activity in F/A WT and Cripto KO cells, from two independent Cripto KO ESC clones (KO.1 and KO.2), and found that it was significantly reduced in Cripto KO compared with Control (Fig. 4d). To evaluate whether this failure to undergo metabolic reprogramming leads to the mutant phenotype, we asked whether inhibition of oxidative phosphorylation by the mitochondrial ATP synthase inhibitor oligomycin^32^ could rescue F/A-induced Cripto KO EpiSCs (Fig. 4e–i). Interestingly, F/A-treated Cripto KO ESCs generated tightly packed EpiSCs-like colonies in the presence of oligomycin, with reduced proliferation rates and large areas of Claudin6-positive cells (Fig. 4e–g). Furthermore, oligomycin treatment fully rescued lactate production (Fig. 4i) and induced the expression of EpiSCs markers (Fig. 4f,h), thus demonstrating that Cripto-dependent metabolic reprogramming is crucial to induce an EpiSC state. To get mechanistic insights into how Cripto acts to regulate this metabolic reprogramming, we focused on the Activin/Nodal/Pgcb axis, which has been recently identified as key regulator of this metabolic switch^31^. Specifically, Activin/Nodal signalling actively represses mitochondrial genes' expression/activity through inhibition of *Pgc-1b*^31^,^33^. Given the key role of Cripto as positive Nodal co-receptor^34^, we hypothesized that a Cripto/Nodal/Pgcb axis may control mitochondrial gene expression/activity and eventually regulate the metabolic reprogramming in ESC→EpiSC transition. In line with this hypothesis, Smad2 phosphorylation was reduced in F/A Cripto KO cells compared with Control (Fig. 4j), whereas *Pgc-1b* expression was significantly upregulated (Fig. 4k). We then went on and knocked down *Pgc-1b* expression in Cripto KO ESC→EpiSC transition by transient transfection of small interfering RNAs at the same time point as in oligomycin treatment (Fig. 4e). Remarkably, *Pgc-1b* downregulation (Fig. 4l,m) repressed *Cox7a1* mitochondrial gene expression (Fig. 4n) and increased lactate production (Fig. 4o). All together, these results provide unprecedented evidence that Cripto regulates the metabolic reprogramming that is crucial for ESC to EpiSC conversion and suggest that it occurs, at least in part, through activation of the Nodal/Pgc-1b/mitochondrial genes axis.

### Cripto deficiency attenuates ESC lineage restriction

**Figure 5:**
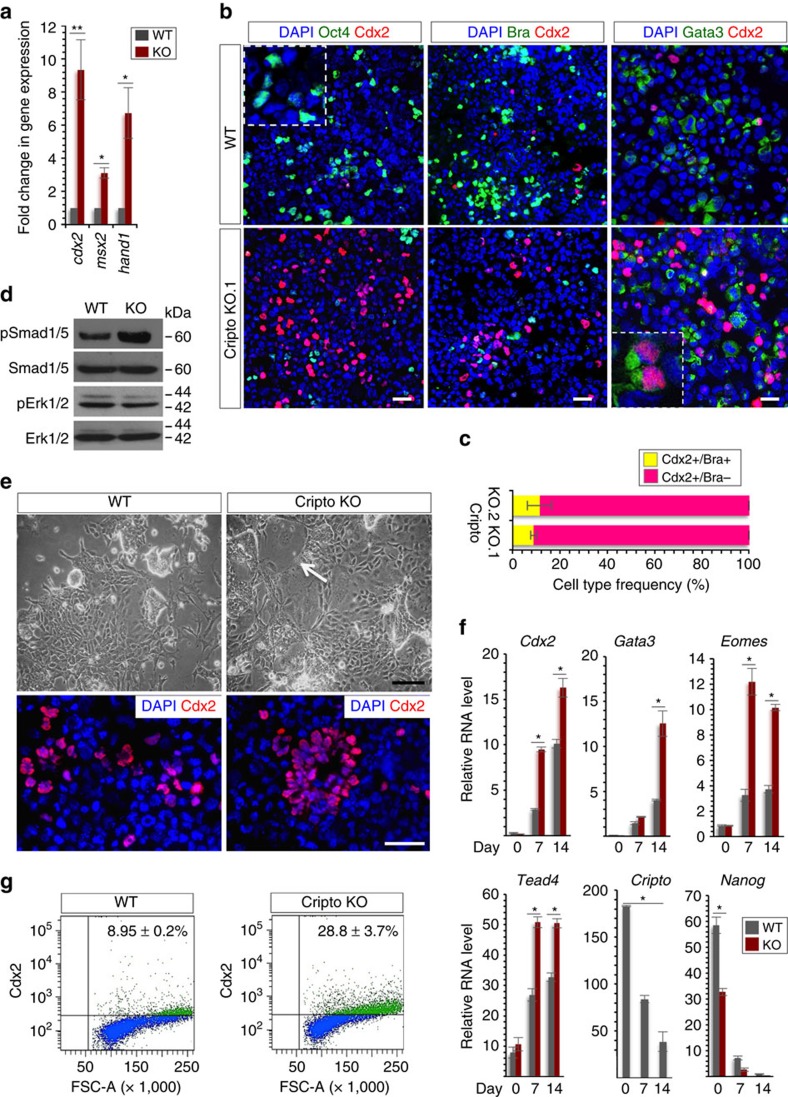
Cripto depletion attenuates ESC differentiation potential towards embryonic lineages *in vitro*. (**a**) qPCR analysis of *Cdx2*, *Msx2* and *Hand1* trophoblast markers in WT and Cripto KO ESC→EpiSC transition (day 6). Data are shown as fold change compared with ESCs after normalization to *Gapdh* and are mean±s.e.m. (*n*=3; *P<0.01). (**b**) Representative pictures of Oct4/Cdx2, Bra/Cdx2 and Gata3/Cdx2 double immunostaining of F/A WT and Cripto KO (clone KO.1) cytospinned cells. Inserts are higher magnification images of selected areas. Nuclei were stained with 4,6-diamidino-2-phenylindole (DAPI). Scale bar, 75 μm. (**c**) Quantification of Cdx2+/Brachyury± cells distribution. Data are expressed as percentage over total number of Cdx2+ cells (*n*≈300) on two independent Cripto KO ESC clones (KO.1 and KO.2) and are mean±s.e.m. (*n*=3). (**d**) Western blot analysis of pSmad1/5 and pErk1/2 protein levels in F/A WT and Cripto KO cells. Smad1/5, Erk1/2 were used as loading controls. (**e**) Photomicrographs of TSC colonies (top panels) and Cdx2 immunostaining in cytospinned cells (bottom panels) (scale bar, 75 μm) generated by WT and Cripto KO ESCs. White arrow indicates a trophoblastic giant cell (scale bar, 100 μm). (**f**) qPCR analysis of TE and pluripotency markers at different time points of WT and Cripto KO TSC differentiation. Relative RNA level was normalised to *Gapdh*; data are mean±s.e.m. (*n*=3; P*<0.01). (**g**) FACS-based quantification of Cdx2-positive cells at 2 weeks of ESC to TSC differentiation. Data are mean±s.e.m. (*n*=3).

Our findings that Cripto is required for ESC→EpiSC transition leave open the question of the fate of F/A-induced Cripto KO ESCs. To address this issue, we first analysed the ∼700 genes that were uniquely deregulated in Cripto KO ESC→EpiSC transition (Fig. 4a). Interestingly, GO analysis revealed a consistent over-representation of genes in the Kyoto Encyclopedia of Genes and Genomes (KEGG) pathway related to pattern specification processes/formation (Supplementary Fig. 3a). Strikingly, this includes a large set of *Hox* genes that were uniquely upregulated in Cripto KO (Supplementary Fig. 3b,c), indicating cell differentiation. Furthermore, we focused our attention on *Cdx2* and *Hand1* genes (Fig. 3e), which are involved in trophoblast lineage specification and differentiation with *Msx2* (ref. 17), and first confirmed that they were all significantly upregulated in Cripto KO transition by qPCR analysis (Fig. 5a)^17^. In line with RNA expression data, the percentage of Cdx2-positive cells almost doubled in F/A Cripto KO cells compared with Control as indicated by FACS analysis (Cdx2^+^ cells 2.6±0.9% WT versus 14.5±3.1% KO; Supplementary Fig. 3d). To further characterize these Cdx2-positive cell population, we performed double staining with Cdx2 and various markers on two independent Cripto KO ESC clones (KO.1 and KO.2). First, consistent with the idea that *Cdx2* plays a central role in blastocyst development by repressing the pluripotency gene *Oct4* (ref. 17), Cdx2-positive cells did not express Oct4 (Fig. 5b and Supplementary Fig. 3e). To assess whether Cdx2 was labelling posterior mesoderm rather than TE^35^, we performed double staining with Cdx2 and Brachyury (Fig. 5b and Supplementary Fig. 3e). Interestingly, quantification of Cdx2+/Brachyury± cells showed that the majority of Cdx2-positive cells did not express Brachyury (Fig. 5c). Finally, consistent with the idea that Cripto deficiency was promoting an extraembryonic fate, we found that Cdx2-positive cells also expressed the trophoblast marker Gata3 (ref. 36, Fig. 5b and Supplementary Fig. 3e). Interestingly, it has been recently shown that Smad2 represses autocrine bone morphogenetic protein (BMP) signalling, which eventually leads to TE differentiation^37^. Remarkably, while Smad2 phosphorylation was reduced in F/A Cripto KO cells compared with Control (Fig. 4j), BMP-dependent Smad1/5 phosphorylation was induced (Fig. 5d), suggesting that this is the mechanism underlying Cripto deficiency-induced TE differentiation. On the contrary, extracellular signal-regulated kinase (ERK) signalling was unaffected (Fig. 5d). To further determine the differentiation capacities of Cripto KO ESCs towards the trophoblast lineage, we subjected WT and Cripto KO ESCs to culture conditions that favour trophoblast stem cell (TSC) differentiation^38^. Both WT and Cripto KO ESCs developed dense undifferentiated colonies (Fig. 5e) and expression analysis revealed a strong upregulation of trophoblast lineage determinants *Cdx2*, *Gata3*, *Eomes*, *Tead4* and *Elf5*, which were significantly higher in Cripto KO versus WT TSC cultures. Conversely, expression of the pluripotency genes *Cripto*, *Oct4* and *Nanog* was strongly reduced (Fig. 5f and Supplementary Fig. 3f). The upregulation of Cdx2 and Eomes in Cripto KO cultures was confirmed by immunofluorescence (Fig. 5e and Supplementary Fig. 3g) and FACS analysis (Cdx2^+^ cells 8.9±0.2% WT versus 28.8±3.7% KO; Fig. 5g). All together, these results indicated that TSC differentiation was induced more efficiently in Cripto KO ESCs. Intriguingly, cells with a morphology characteristic of trophoblast giant cells were observed in Cripto KO but not in WT cultures (Fig. 5e), leading to speculate that these cells were undergoing spontaneous trophoblast differentiation. Interestingly, this is consistent with previous findings that Nodal is required to sustain the TSC stemness and inhibit their precocious differentiation *in vivo*^39^.

**Figure 6:**
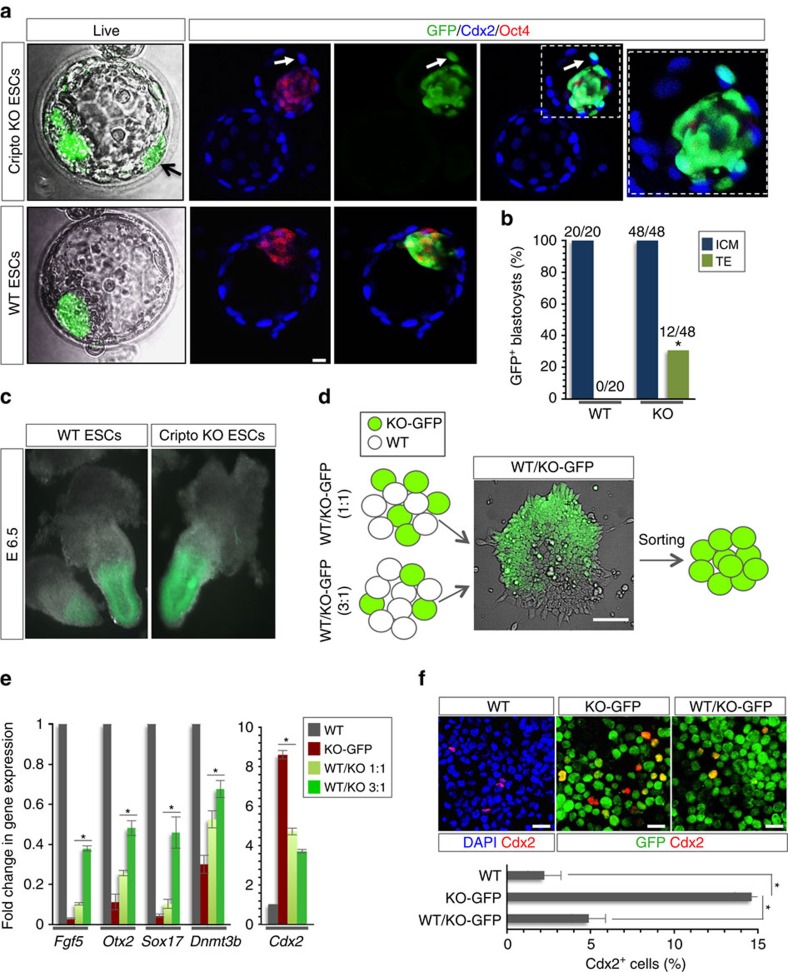
Cripto depletion attenuates ESC differentiation potential towards embryonic lineages *in vivo.* (**a**) Chimeric blastocysts from GFP-labelled WT and Cripto KO ESCs by live confocal microscopy imaging (original magnification × 20) and whole-mount immunostaining against GFP, Cdx2 and Oct4; arrow marks GFP^+^ TE cells (scale bar, 10 μm). (**b**) Frequency of blastocysts with GFP^+^ ICM and TE cells from WT (blastocysts *n*=20) and Cripto KO (blastocysts *n*=48) ESCs. Fisher's exact test *P<0.05. (**c**) Representative pictures by AXIOZoom.V16 Zeiss microscopy (original magnification × 10) of chimeric embryos from GFP-labelled WT and Cripto KO ESCs injected into morula and dissected at E6.5. (**d**) Schematic representation of the experimental procedure and representative picture of co-culture WT and Cripto KO colony. (**e**) Effect of F/A on the expression of selected markers in WT, Cripto KO ESCs and WT ESCs mixed at 1:1 and 3:1 ratios with Cripto KO-GFP ESCs. Relative RNA level was normalized to *Gapdh*; data are mean±s.e.m. (*n*=3; P*<0.01). (**f**) Representative pictures of 4,6-diamidino-2-phenylindole (DAPI)/Cdx2 and GFP/Cdx2 double immunostaining (upper panels; scale bar, 75 μm) and quantification of Cdx2+ cells (bottom panel; ∼100 cells per condition) of F/A WT, Cripto KO and WT/Cripto KO cytospinned cells mixed at 3:1 ratio. Data are mean±s.e.m. (*n*=3; P*<0.01).

To further explore this phenotype, we assessed the capacity of Cripto KO ESCs to contribute to TE *in vivo*. To this end, green fluorescent protein (GFP)-labelled WT and Cripto KO ESCs were microinjected into morulas and the resulting blastocysts were examined. Both WT and Cripto KO ESCs efficiently contributed to the ICM. Interestingly, Cripto KO ESCs also colonized the TE^40^, whereas, as expected, WT ESCs did not contribute to this extraembryonic lineage (Fig. 6a,b). Accordingly, whole-mount immunostaining for the trophoblast marker Cdx2 and the ICM marker Oct4 showed that Cripto KO-GFP ESCs in the TE expressed Cdx2, whereas GFP co-localized with Oct4 in the ICM of both WT and Cripto KO chimeric blastocysts (Fig. 6a). To further assess the developmental plasticity of Cripto KO ESCs in the embryonic context and evaluate their contribution to the extraembryonic tissue, injected morulas were implanted into foster mothers and chimeric embryos were analysed at E6.5 (Fig. 6c). None of the scored Cripto KO-GFP chimeric embryos (*n*=25) showed contribution to the extraembryonic ectoderm or to the ectoplacental cone; however, they showed contribution to the EPI and were phenotypically WT (Fig. 6c). Of note, we hypothesized that Cripto KO cells have a propensity to rapidly differentiate into giant cells (Fig. 5e) and this could explain why these cells cannot be maintained within the chimeras. This result can also be explained by a non-cell autonomous contribution of Cripto in the WT environment^41^. To address this hypothesis, WT ESCs were mixed at different ratios with Cripto KO-GFP ESCs (1:1 and 3:1). Interestingly, at each ratio F/A-treated Cripto KO ESCs generated tightly packed EpiSCs-like colonies similar to that of WT F/A EpiSCs, suggesting that co-culture with WT ESCs rescued the mutant phenotype (Fig. 6d). To further explore this phenotype, F/A Cripto KO–GFP cells were flow-sorted by using the GFP reporter (Supplementary Fig. 4) and assayed separately for EpiSC and TE factors. In line with our hypothesis, expression of EpiSC markers was induced in a dose-dependent manner, concomitant with a downregulation of *Cdx2* expression (Fig. 6e), which was confirmed by immunofluorescence (Fig. 6f). All together, our data support a model wherein Cripto deficiency affects ESC→EpiSC transition and attenuates the normal restriction of ESCs towards embryonic tissue.

### Cripto/Nodal sustains mEpiSC and hESC self-renewal

**Figure 7:**
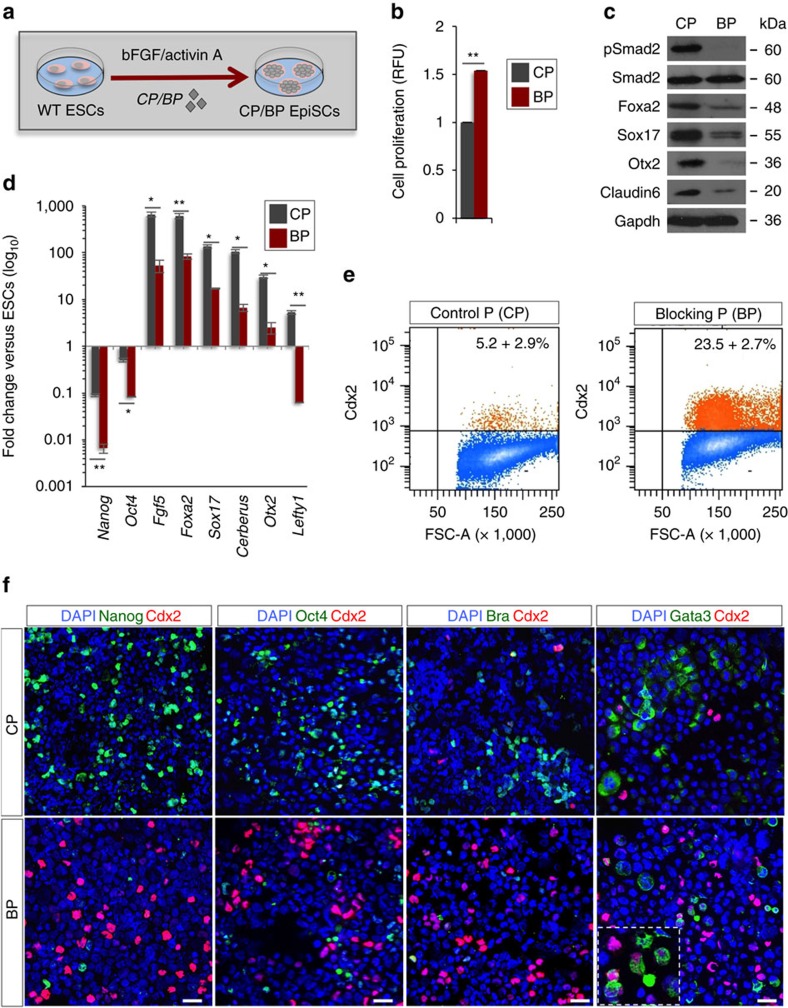
Pharmacological inhibition of Cripto signalling prevents ESC→EpiSC transition. (**a**) Schematic representation of the experimental procedure. (**b**) Proliferation of CP/BP-treated cells measured by the CyQuant assay and expressed as RFU. (**c**) Western blot analysis of pSmad2, Foxa2, Sox17, Otx2 and Claudin6 in CP/BP-treated F/A cells. Smad2 and Gapdh were used as a loading control. (**d**) Gene expression profiles of selected markers in CP/BP-treated F/A EpiSCs. Data are shown as fold change compared with ESCs (serum/LIF) after normalization to *Gapdh* and are mean±s.e.m. (*n*=3; *P<0.01 and **P<0.005). (**e**) FACS quantification of Cdx2^+^ cells in CP/BP-treated cultures. Data are mean are mean±s.e.m. (*n*=3). (**f**) Representative pictures of Nanog/Cdx2, Oct4/Cdx2, Bra/Cdx2 and Gata3/Cdx2 double immunostaining of CP/BP-treated cytospinned cells (scale bar, 75 μm). Inserts are higher magnification images of selected areas. Nuclei were stained with 4,6-diamidino-2-phenylindole (DAPI).

To get further mechanistic insights into the role of Cripto in the establishment of primed pluripotency, we exploited the activity of a Cripto/ALK-4 blocking peptide (BP), which binds Cripto and antagonizes Cripto/ALK-4 interaction^42^ (Fig. 7a). Although control peptide (CP)-treated F/A ESCs developed EpiSCs-like colonies, Cripto BP-treated cells raised colonies containing highly proliferating and morphologically heterogeneous cell populations (Fig. 7b). As expected, Smad2 phosphorylation was reduced in Cripto BP-treated cells (Fig. 7c), concomitant with the downregulation of EpiSC markers and the pluripotency genes (Fig. 7c,d), as well as upregulation of TE markers (Supplementary Fig. 3h). Furthermore, similar to that observed in F/A-induced Cripto KO cells, Cdx2-positive cells strongly increased in the presence of Cripto BP (Fig. 7e,f). Interestingly, these Cdx2-positive cells did not express Oct4 and Nanog, and few of them stained positive for the mesoderm marker Brachyury, which in turn was downregulated (Fig. 7f). Finally, we found co-expression of Cdx2 with the trophoblast marker Gata3. All together, these data demonstrate that Cripto/ALK-4/Nodal signalling is required for ESC→EpiSC transition and to restrict ESC differentiation potential towards embryonic tissues.

**Figure 8:**
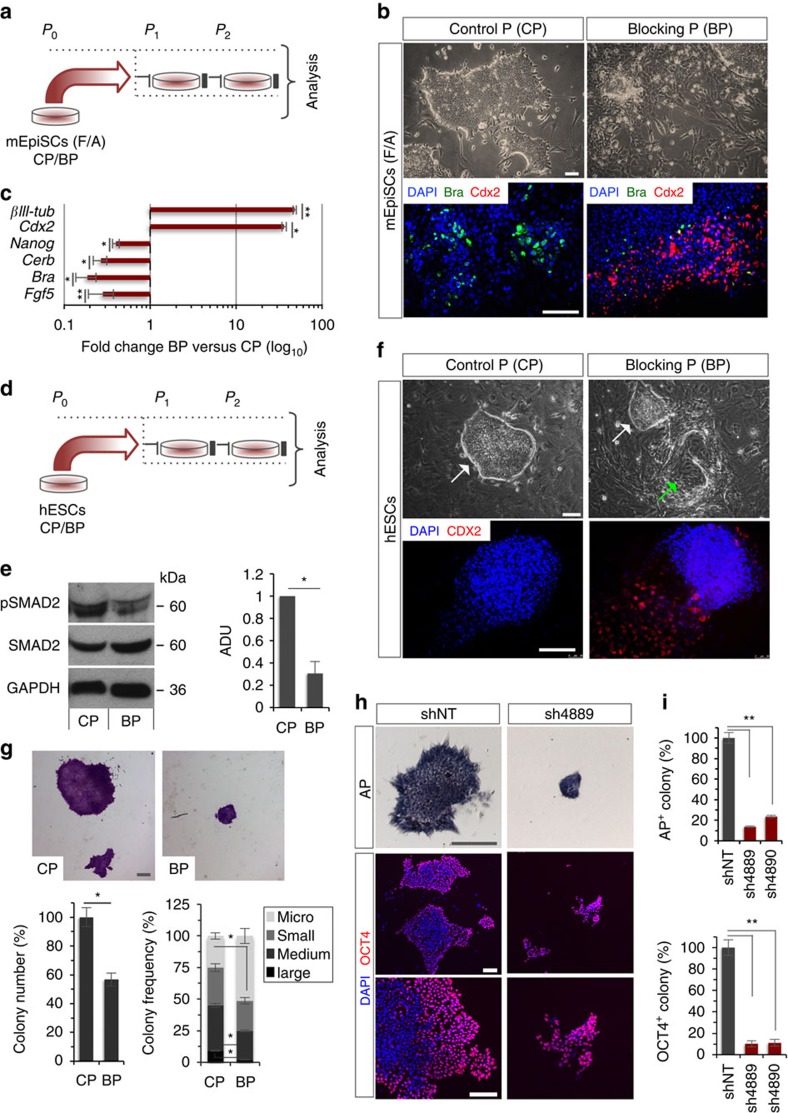
Cripto is required for maintenance of mouse and human primed pluripotent stem cell states. (**a**) Schematic representation of the experimental procedure. (**b**) Representative photomicrographs of brightfield (scale bar, 100 μm) CP/BP-treated F/A EpiSCs colonies (top panels) and immunostaining detection of Bra/Cdx2 (bottom panels) in F/A EpiSCs, after two passages with CP/BP. Nuclei were stained with 4,6-diamidino-2-phenylindole (DAPI). (**c**) qPCR analysis of EPI -associated (*Fgf5*, *Brachyury* (*Bra*), *Cerberus* (*Cer*) and *Nanog*), trophoblast (*Cdx2*) and neural (*βIII-tubulin*) markers in BP/CP-treated F/A mEpiSCs. Data are expressed as fold change of BP versus CP after normalization to *Gapdh* and are mean±s.e.m. (*n*=3; *P<0.01 and **P<0.005). (**d**) Schematic representation of the experimental procedure. hESCs were passaged either on feeder layer or in Matrigel/feeder-free conditions and in the presence of CP/BP. (**e**) Western blot analysis of pSMAD2 protein levels in CP/BP-treated hESCs (1 week). SMAD2 and GAPDH were used as loading controls. The densitometric analysis is expressed in arbitrary unit (ADU) as the pSMAD2/SMAD2 ratio. (**f**) Representative phase contrast pictures (upper panels) of CP/BP-treated hESC colonies after two passages on feeder layer. White and green arrows indicate undifferentiated and differentiated colonies, respectively. Representative picture of CDX2 immunostaining (lower panels) in CP- and BP-treated hESCs. Nuclei were stained with DAPI. Scale bars, 100 μm. (**g**) Clonogenic assay of hESCs treated with CP and BP for two passages on Matrigel-coated plates. Representative pictures of CP- and BP-treated hESC colonies, stained with crystal violet (top panels). Scale bar, 200 μm. Colony number (bottom left panel) and phenotype frequency (bottom right panel). Data are mean±s.e.m. (≈100 colonies scored/condition; *P<0.01). (**h**) Representative photomicrographs of hESC colonies derived from shNT Control and sh4889 *CRIPTO* KD hESCs, and stained for AP (top panels; scale bar, 200 μm) or OCT4 (bottom panels; scale bar, 100 μm). (**i**) Quantification of AP+ (top panel) and OCT4+ (bottom panel) colonies. The number of AP+ and OCT4+ colonies is shown as percentage over shNT hESCs. Data are mean±s.e.m. (*n*=3; **P<0.005).

We then asked whether Cripto signalling was required to maintain EpiSC self-renewal. To this end, F/A WT EpiSCs were analysed after subsequent passages in culture in the presence of Cripto CP or BP (Fig. 8a). Although CP-treated EpiSCs developed homogeneous cell colonies with a typical flat morphology, Cripto BP-treated EpiSCs colonies progressively lose their characteristic morphological appearance and become differentiated (Fig. 8b). Accordingly, EpiSCs markers were strongly downregulated, whereas expression of the neuronal differentiation marker βIII-tubulin was induced (Fig. 8). Interestingly, Cdx2 expression was also strongly induced in BP-treated EpiSCs and the majority of Cdx2+ cells did not express Brachyury (Fig. 8b,c). Together, these findings indicated that Cripto-dependent Nodal signalling is required to sustain EpiSCs pluripotency/self-renewal and prompted us to extend the analysis to hESCs, which are similar to mEpiSCs. Consistent with what was previously reported^43^, surface CRIPTO protein was highly expressed in hESCs (Supplementary Fig. 5a). To investigate whether CRIPTO is required to maintain hESC pluripotency, we evaluated the effect of blocking CRIPTO signalling. To this end, hESCs were grown on feeders in the presence of either Cripto BP or CP and analysed after two passages (Fig. 8d). Similar to that observed in F/A EpiSCs, SMAD2 phosphorylation was strongly inhibited in Cripto BP-hESCs, already after the first passage in culture, thereby proving the activity of the BP (Fig. 8e). Interestingly, although CP-hESCs maintained their undifferentiated morphology, Cripto BP-hESC colonies appeared heterogeneous and clearly showed areas of differentiated cells that stained positive for CDX2 (Fig. 8f). Consistent with the idea that SMAD2 represses autocrine BMP signalling, which in turn induces CDX2-positive trophoblast committed cells^37^,^44^, and in line with our findings on mEpiSCs (Fig. 5d), BMP-dependent SMAD1/5 phosphorylation was induced in Cripto BP-hESCs (Supplementary Fig. 5b). To rule out the possibility that these cells may represent a subpopulation of mesodermal cells that go through a BRACHYURY-positive state^45^, we performed double CDX2/BRACHYURY staining and found only few BRACHYURY/CDX2 double-positive cells (Supplementary Fig. 5c). We thus went on and evaluated the biological effect of Cripto BP on hESC self-renewal. Remarkably, we found a dramatic decrease of the colony-formation capacity of Cripto BP-hESCs compared with CP in semisolid cultures (Fig. 8g). Moreover, Cripto BP-hESC colonies were significantly smaller in size (Fig. 8g), thus indicating that blocking CRIPTO signalling affected hESC self-renewal potential. Furthermore, poly (ADP-ribose) polymerase (PARP) activation was detected in Cripto BP-hESCs, suggesting increased cell death (Supplementary Fig. 5d). All together, these data point to a key role of CRIPTO signalling in sustaining hESC pluripotency and self-renewal, and preventing their transdifferentiation into extraembryonic derivatives. To validate the findings obtained by pharmacological inhibition of CRIPTO, we assessed the effect of *CRIPTO* silencing using lentiviral vectors containing two different short hairpin (shRNA) sequences targeting the 3′-untranslated region and the coding sequence of *CRIPTO*, respectively^23^. Silencing of *CRIPTO* by lentiviral shRNA knockdown (KD) resulted in a strong reduction of CRIPTO protein expression (Supplementary Fig. 5e). CRIPTO KD and Control hESCs were then subjected to a matrigel clonogenic assay and the resulting colonies were analysed by immunofluorescence for the pluripotency markers OCT4 and NANOG, and AP staining. The quantification analysis of either OCT4-, NANOG- or AP-positive colonies showed a dramatic reduction of colony number in both *CRIPTO* KD hESC clones, which were also significantly smaller in size compared with Control (Fig. 8h,i and Supplementary Fig. 5f–h), thus providing evidence that CRIPTO KD strongly affected the clonogenic potential of hESCs.

All together, these findings indicate that CRIPTO sustains hESC self-renewal, at least in part, through activation of NODAL signalling.

## Discussion

**Figure 9:**
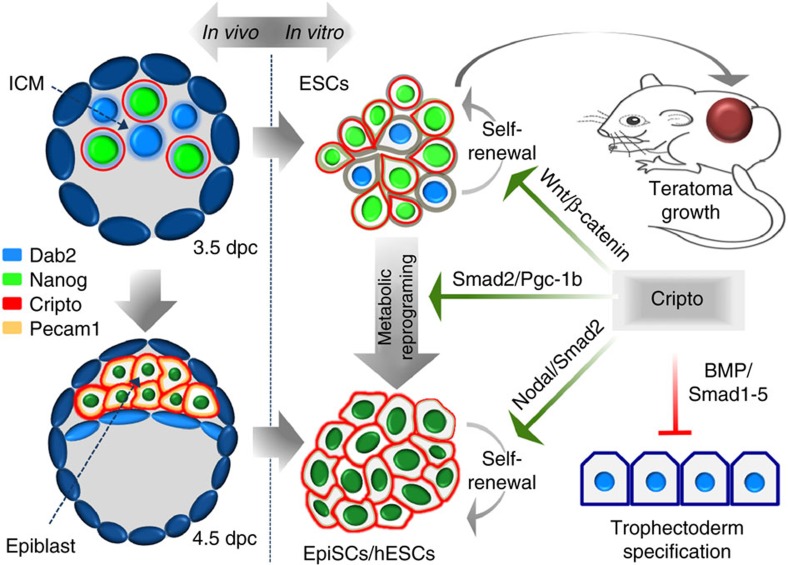
Schematic summary of Cripto activity in naive and primed pluripotent stem cell states. Cripto is one of the earliest EPI markers. Cripto-positive cells display a salt-and-pepper distribution and co-localize with Nanog-positive cells (light green) within the ICM of the pre-implantation blastocyst (E3.5). Next, *Cripto* expression becomes homogeneous in the late blastocyst (E4.5), marking all the cells of the EPI (dark green), whereas it is absent in both the TE (dark blue) and in the PrE (light blue). In mouse ESCs, surface Cripto is heterogeneous and highly dynamic and correlates with high levels of pluripotency markers. Moreover, Cripto regulates mouse ESC self-renewal by positively modulating the canonical Wnt/β-catenin pathway. Conversely, Cripto regulates the metabolic reprogramming that occurs in the transition from ESCs to EpiSCs, at least in part through the Nodal/Smad2/Pgc-1b axis. Finally, Cripto/Nodal/Smad2 sustains mouse EpiSC/hESC self-renewal and prevents transdifferentiation towards the trophoblast lineage, by repressing BMP/Smad1-5 signalling. Our findings place Cripto at the interface of the mouse and human pluripotency networks, and provide unprecedented evidence that it restricts ESC differentiation potential towards embryonic tissue.

This work shows that the membrane protein Cripto is essential for mouse EpiSC and hESC pluripotency, and provides mechanistic insights into how the extracellular environment controls early cell-fate decisions in the embryo (Fig. 9). We demonstrate that Cripto is one of the earliest EPI markers and provide unprecedented evidence that it plays a pivotal functional role in the acquisition/maintenance of mouse and human pluripotency. Consistent with the salt-and-pepper distribution of Cripto within the ICM of the pre-implantation blastocyst, which underlies the emergence of EPI and PrE lineages^1^, surface Cripto protein is heterogeneous and highly dynamic in serum/LIF ESCs and positively correlates with the expression of the pluripotency factors. Heterogeneity and fluctuations in the expression of pluripotency markers in ESCs may be considered as culture-induced perturbations and their relevance to pluripotency and cell-fate decision is still a matter of debates^46^. Our *in vivo* and *in vitro* results challenge the idea of a functional role of Cripto heterogeneity in pluripotency. Indeed, *Cripto* genetic ablation facilitates the exit from naive pluripotency *in vitro* and affects both the efficiency and latency of ESC-derived teratomas formation. At mechanistic level, Cripto regulates mouse ESC self-renewal by positively modulating the canonical Wnt/β-catenin pathway. Despite the fact that Cripto deficiency facilitates the exit from naive pluripotency in FBS/LIF ESCs, this effect does not accelerate the transition to the primed state, but rather affects the conversion of ESCs to EpiSCs. Remarkably, this Cripto KO mutant phenotype is due to impaired metabolic switch from OXPHOS to aerobic glycolysis, which is required to convert ESCs to EpiSCs^31^. Indeed, while mouse ESCs are bivalent in their energy production, dynamically switching from glycolysis to mitochondria respiration, both mEpiSCs and hESCs are highly glycolytic^31^. Our findings point to a functional link between Cripto and glycolytic metabolism *in vitro* and raise the intriguing possibility that it may play a similar role *in viv*o. Indeed, it has been recently proposed that Activin/Nodal controls the proper metabolic switch in early embryo by repressing mitochondrial activity through inhibition of PGC-1b^31^. In line with this idea and according to the key role of Cripto as Nodal coreceptor, *Pgc-1b* is upregulated in F/A Cripto KO cells and its downregulation is able to increase lactate production. Although additional experiments are needed to get further insights into the mechanism, our data suggest that Cripto controls the metabolic reprogramming, at least in part, through the Nodal/Pgcb axis. Consistent with the idea that this metabolic switch is a key event in ESC→EpiSC transition^31^, we demonstrate that Cripto is required to generate/maintain FGF/Activin (F/A) EpiSCs. Indeed, we cannot rule out the possibility that Cripto KO F/A EpiSCs form but rapidly and continuously get lost from the cultures and/or differentiate. However, quite unexpectedly, we find that Cripto deficiency skews ESC differentiation towards TE *in vitro*. Several data support this intriguing idea. TE markers are induced at RNA and protein level; specifically, Cdx2 significantly increased in F/A Cripto KO cells and does not co-localize with Brachyury, thus ruling out the possibility that Cdx2-expressing cells identify posterior mesoderm rather than TE. Furthermore, Cdx2 does not co-localize with Oct4, consistent with the mutually exclusive expression of these genes during embryogenesis^17^. Finally, Cdx2-positive cells also express the TE maker Gata3 (ref. 36). Concomitant to induction of TE markers, expression of the DNA methyltransferase *Dnmt3b* is reduced in F/A Cripto KO cells. Interestingly, *Dnmt*-deficient and therefore hypomethylated ESCs/embryos show transdifferentiation to the extraembryonic trophoblast lineage^47^. In line with these findings, morphological and molecular analyses indicate that Cripto KO ESCs facilitate the generation of TSCs *in vitro*. Interestingly, several evidence indicate that BMP signalling pathway plays a crucial role in TE differentiation of pluripotent stem cells. Specifically, *Cdx2* expression is directly regulated by the BMP–Smad1/5 pathway^48^, which in turn is repressed by Smad2 (ref. 37). Accordingly, *Cripto* KO inhibits Smad2 activation and conversely induces BMP-dependent Smad1/5 phosphorylation, suggesting that derepression of BMP signalling may account for TE differentiation of F/A-induced Cripto KO cells. Most remarkably, according to the idea that *Cripto* deficiency attenuates the normal restriction of mouse ESCs to embryonic lineages, Cripto KO ESCs gain the unique property to colonize TE in blastocyst chimera, which is absent in WT ESCs^40^. However, analysis of E6.5 chimeric embryos generated with Cripto KO ESCs indicates that these cells efficiently contribute to the embryonic but not to the ectoplacental cone. Although we cannot rule out the possibility that Cripto KO ESCs that colonize the TE fail to differentiate properly and thus to efficiently contribute to the trophoblast derivatives, we speculate that a non-cell autonomous activity of Cripto in the WT environment may rescue, at least in part, the mutant phenotype. Although further investigations are required to directly address this issue, this hypothesis is consistent with co-culture data showing that non-cell autonomous Cripto is able to rescue F/A Cripto KO EpiSCs. Intriguingly, to date, no defects in EPI specification/maintenance have been reported in *Cripto*-null mutants that indeed are defective in anterior–posterior axis formation^14^. Of note, data in different mutant context indicate that embryo can tolerate a significant loss of EPI cells^1^, thus suggesting that a mild phenotype, such as a smaller EPI, could be rescued by the plasticity of the embryo. In this respect, embryonic development might be less vulnerable than ESC differentiation, as it relies on a more complex network of signals easier to compensate than in ESCs, which may reconcile the apparent discrepancy. Moreover, Cripto overlooked function(s) *in vivo* can be also explained by the activity of the epidermal growth factor (EGF)-CFC *Cryptic*. Indeed, *Cryptic* promotes EPI maintenance in the absence of *Cripto* presumably through a non-cell-autonomous activity, as it is only expressed in the visceral endoderm^19^. Finally, we speculate that a maternal contribution of Cripto may also compensate for the absence of Cripto in the early embryo. Accordingly, recent findings report Cripto expression in the endometrium during pregnancy, and in the mouse uterus^49^.

All together, our findings have important implications for stem cell biology and for our understanding of the mechanisms that regulate TE segregation from the ICM. First, existing models that Activin/Nodal signalling sustains mEpiSC generation/self-renewal mainly rely on the effect of the small molecule SB431542 (ref. 31), which however is a general antagonist of the TGFβ pathway^50^. Here we overcome this limitation and provide evidence that Cripto/Nodal/Smad2 is required for mEpiSC generation and self-renewal, and moreover that it is functionally conserved in human pluripotent stem cells. Indeed, consistent with the idea that SMAD2 represses autocrine BMP signalling, which conversely induces TE differentiation in hESCs^37^,^44^, pharmacological blocking of Cripto/SMAD2 results in increased BMP-dependent SMAD1/5 signalling and TE differentiation.

In summary, our studies provide unprecedented evidence that Cripto is a major determinant of mEpiSCs/hESCs pluripotency and add novel important elements to the current model of mammalian pluripotency. Moreover, our findings suggest that *Cripto* may have earlier functions than previously recognized, in the very first lineage decision made by the early embryo.

## Methods

### Blastocyst collection and whole-mount *in situ* labelling

Embryos were produced by natural matings of CD1 mice. For immunostaining, embryos were fixed with 4% paraformaldehyde (PFA) in PBS overnight at 4 °C, permeabilized with 0.5% Triton X-100 in PBS for 15 min and blocked in 10% FBS in PBS-0.1% Triton for 1 h^51^. Fluorescent *in situ* hybridization was described in ref. 52. Experiments were performed in accordance with French and EU guidelines for the care and use of laboratory animals. List of antibodies are in Supplementary Table 1.

### Mouse ESC culture and EpiSC generation and maintenance

WT and Cripto KO R1 mESCs were cultured in high glucose DMEM medium (Invitrogen, Life Technologies) supplemented with 15% ES-screened FBS (Euroclone), 0.1 mM β-mercaptoethanol (Sigma-Aldrich), 1 mM sodium pyruvate, 2 mM glutamine, 100 U ml^−1^ penicillin/streptomycin (all from Gibco) and 1,000 U ml^−1^ recombinant LIF (ESGRO, Millipore)^15^. 2i Medium was supplemented with PD0325901 (1 μM) and CHIR99021 (3 μM)^6^. Two independent Cripto KO ESC clones, that is, DE7 and DE14 (named KO.1 and KO.2), were used throughout the study and were previously described^15^,^28^. For stimulation with bFGF, Activin A and Bmp4, 2 × 10^6^ ESCs were plated in N2B27 on serum-coated dishes and incubated (15′) with bFGF (12 ng ml^−1^, Provitro) or Bmp4 (50 ng ml^−1^, R&D). Cells were LIF starved (6 h) before stimulation with LIF.

EpiSCs were generated from ESCs as described^5^. Briefly, WT and Cripto KO ESCs were seeded at low density (3 × 10^3^ cells per cm^2^) in N2B27 supplemented with Activin A (20 ng ml^−1^, Invitrogen) and bFGF, and cultured for 6 days.

Cripto BP and CP were dissolved in dimethylsulfoxide and media with peptides were refreshed every other day during ESC to EpiSC transition.

### Colony-forming assay

For colony assay, ESCs were trypsinized to obtain a single-cell suspension and plated at low density (100 cells per cm^2^) in the culture conditions described. After 6 days, colonies were fixed in 4% PFA and stained with crystal violet. Briefly, cells were washed twice with PBS and fixed/stained with a solution of 6% glutaraldehyde and crystal violet. After 30 min at room temperature, cells were carefully washed with tap water and dried for further analysis. Images were collected on a DMI6000B microscope (Leica Microsystems). The morphological classification (domed/flat) was performed blinded by two investigators. Experiments were performed in triplicate.

### Proliferation assays and viable cell count

Cell proliferation was measured using the colorimetric CyQuant cell proliferation assay (Invitrogen), following the manufacturer's instructions. Absorbance was analysed at 480–520 nm, using the Fluoroskan Ascent FL Microplate Fluorometer and Luminometer (Thermo Fisher Scientific, Waltham, MA, USA).

### Trophoblast stem cell (TSC) culture

To generate TSCs, ESCs were cultured in TCS medium containing RPMI 1640, 20% FBS (Thermo Scientific), 25 ng ml^−1^ FGF-4 (Invitrogen), 1 ng ml^−1^ Heparin (Sigma-Aldrich), 2 mM L-glutamine, 0.1 mM β-mercaptoethanol, 1 mM sodium pyruvate and 50 units per ml^1^ Penicillin/Streptomycin (Invitrogen), with 70% feeder conditioned media^38^. ESCs were plated in TSC medium on gelatin-coated plates in the absence of feeders and passaged every 3 days, and the medium changed every 2 days.

### hESC culture and self-renewal assay

The hESC line CSES7 (NIH registration number: 0107) was used throughout the study and was kindly provided by Professor Nissim Benvenisty (The Hebrew University, Israel). Undifferentiated hESCs were maintained in culture either on feeder layer or under feeder-free conditions, using standard procedures. For culture on feeder layer, hESCs were grown in Knockout DMEM (Gibco), supplemented with 20% Knockout Serum Replacement (Gibco), 2 mM glutamine, 1 × non essential amino acids (NEAAs), 100 U ml^−1^ Penicillin/Streptomycin, 0.1 mM 2-Mercaptoethanol, 10 ng ml^−1^ bFgf (Gibco). For growth under feeder-free conditions, hESCs were cultured on Matrigel (BD Bioscience)-coated plates, using mTeSR medium (Stem Cell Technologies). hESCs were passaged every 5/6 days. Cells were incubated with TrypleSelect 1 × (Gibco) for 2/3 min and scraped gently with a tip.

For clonogenic assay, hESCs were dissociated with TrypleSelect 1 × , for 5 min at 37 °C. Dissociated cells were plated on Matrigel and cultured in mTeSR medium in the different conditions. After 6 days in culture, colonies were dissociated and replated at low density in the same conditions. After 4 days, colonies were fixed and stained either with crystal violet (see above for details) or with the Alkaline Phosphatase kit (System Biosciences) to determine AP activity, following the manufacturer's instructions. The colony number and size were analysed, using ImageJ software. Colonies were classified on the basis of the diameter, as follows: micro <0.2 mm, small 0.2–0.4 mm, medium 0.4–0.7 mm and large >0.7 mm.

For generating *CRIPTO* KD hESCs, lentiviral pLKO.1 vectors containing either a non-targeting sequence or the shRNA4889 and the shRNA4890 (ref. 23) were used. Cells were plated on Matrigel and cultured in mTeSR medium in the different conditions. Colonies were fixed and stained either with AP (System Biosciences) or Oct4 and Nanog antibodies. The colony number was analysed, using ImageJ software. 

Cripto BP and CP were used at 10 and 20 μM in hESC passages and clonogenic assay, respectively.

### Flow cytometry and cell sorting

Single-cell suspensions of EpiSCs and hESCs were obtained using either trypsin-EDTA or TrypleSelect 1 × (Gibco), fixed, stained with the appropriated primary and secondary antibodies according to the manufacturer's protocols and were either analysed with a FACS Canto (Becton Dickinson) or sorted with a FACSAria (Becton Dickinson).

### Western blotting

Whole-cell lysates were prepared with ice-cold immunoprecipitation assay (RIPA) lysis buffer. For β-catenin localization, subcellular fractionation was performed as described^53^. Detection was performed with ECL reagents (Amersham Biosciences). Densitometric analysis was carried out using the software GelEval 1.35. List of antibodies is in Supplementary Table 1. Full blots are in Supplementary Fig. 6.

### Immunohistochemistry

Samples were processed with the standard streptavidin–biotin–immunoperoxidase method (DAKO Universal Kit, DAKO Corp., Carpinteria, CA, USA). Diaminobenzidine was used as the final chromogen and haematoxylin as the nuclear counter stain. Details and list of antibodies are in Supplementary Table 1.

### Immunofluorescence and cytospin samples preparation

Cells were fixed (4% PFA) and permeabilized (0.1% Triton X-100), where necessary, at room temperature. After incubation with primary antibodies, cells were incubated (1 h) with the appropriate secondary antibodies (Alexa Fluor 488, 594 1:200; Molecular Probes). For preparation of cytospin samples, cells (1–1.5 × 10^4^) were dissociated with accutase for 5 min at 37 °C and resuspended in 15% FBS/1 × PBS. Samples were centrifuged at 900  r.p.m. for 15 min onto glass slides (2 spots, 1 × 10^5^ cells each) using a Thermo Shandon Cytocentrifuge (CytoSpin 4, Thermo Fisher Scientific). Specimens were directly analysed or fixed for further analysis. Details and list of antibodies are in Supplementary Table 1.

### RNA extraction and quantitative reverse transcriptase–PCR

Total RNAs were isolated using RNeasy mini kit and reverse transcribed using QuantiTect Reverse Transcription kit (Qiagen). qPCR was performed using SYBR Green PCR master mix (FluoCycle II SYBR, EuroClone). Details and list of primers are in Supplementary Table 2.

### Whole-genome expression analysis

RNA-seq was performed at the Institute for Applied Genomics using the Illumina HiSeq 2500 platform (http://www.igatechnology.com/). Gene expression analysis was obtained by counting reads mapped on gene features, by using *htseq-count*^54^ and the statistical analysis was performed by using RNASeqGUI^55^. In detail, paired-end fastq files were first aligned on the mouse genome version mm9 (downloaded from http://hgdownload.cse.ucsc.edu/goldenPath/mm9/chromosomes) by using TopHat2 (version tophat-2.0.13)^56^ with -G option and using as gene annotation file the release 67 of NCBIM37, downloaded from ftp://ftp.ensembl.org/pub/release-67/gtf/mus_musculus/ repository; all other parameters were set as default. HTSeq^54^ (version 0.6.1p1) was used to obtain raw counts on intersection non-empty mode by using the same gene annotation file. Next, raw counts were processed by using the RNSeqGUI (version 0.99.3)^55^. In particular, the ‘normalize.quantiles' normalization function of the preprocessCore R package (Bolstad B.M. preprocessCore: A collection of pre-processing functions; version preprocessCore_1.28.0) was used to normalize the gene expression values. Those genes with low expressions in all samples were filtered out by using the ‘Proportion test' of NOISeq^57^ (version NOISeq_2.8.0). In the filtering step we used cpm=1. Finally, we used full quantile-normalized and filtered counts to perform differentially expression analysis. For such purpose, we used NOISeq (version 2.8.0) with posterior probability set to 0.95. As NOISeq test has a stochastic component, for each comparison we launched NOISeq ten times, each time setting a different seed. Hence, for each gene we calculated the mean of the ten posterior probabilities obtained so far. We considered a gene as differentially expressed across the samples if the mean of the ten posterior probabilities was ≥0.95. A sketch of the code used for carrying out the statistical analysis is given in Supplementary Data 4. Despite the fact that we have applied a stringent procedure, the absence of replicates does not allow us to define significance in a rigorous statistical sense. Yet, the RNA-Seq results were validated by independent experiments.

Genes were classified according to their known or predicted biological functions based on GO terms (DAVID Bioinformatics Resources; http://david.abcc.ncifcrf.gov).

### Luciferase reporter assay

ESCs were transfected with the Super8xTOPFlash and the *Renilla*-TK mutant plasmids using Lipofectamine, according to the manufacturer's protocol (Invitrogen). At 24 h after transfection, ESCs were treated with Wnt3a (R&D Systems, 5–50 ng ml^−1^) for 24 h^58^, and Luciferase activity was measured by using a dual Luciferase assay kit (Promega) according to the manufacturer's instructions.

### Lactate activity assay

Lactate was measured using the colorimetric L-Lactate Assay Kit (Abcam, Cambridge, MA, USA; ab65331) according to the manufacturer's instructions. Data were normalized to total cell number.

### Teratoma assay

ESCs were trypsinized into single-cell suspension and resuspended in PBS. ESCs (3 × 10^6^) were injected subcutaneously into hind limbs of severe combined immunodeficiency mice. Teratomas were collected, fixed in 4% PFA, sectioned and stained with haematoxylin/eosin or subjected to immunohistochemistry for the histological analysis.

Experiments were done in accordance to the law on animal experimentation (article 7; D.L. 116/92) under the Animal Protocol approved by the Italian Ministry of Health.

### GFP-labelled ESCs and TE lineage contribution

GFP was inserted in both WT and Cripto KO ESCs at the Rosa26 locus by using the R26P-SA-EGFPpuro plasmid (Addgene). Ten days after transfection, puromycin-selected clones were verified for correct self-renewal and differentiation properties.

Chimeras were obtained by injecting WT and Cripto KO GFP-labelled ESCs (13–16) into 4- to 8-cell-stage embryos using standard techniques. Morulas were incubated 48 h in KSOM (Chemical International) and observed under the confocal microscope (Zeiss LSM700). Chimeric mouse generation was performed by morula injection of WT and Cripto KO GFP-labelled ESCs. Resultant embryos were cultured for 48 h *in vitro* and implanted by uterus transfer into pseudopregnant foster mothers using standard methods. Pregnant mice were killed at day E6.5 and whole embryos were photographed with ApoTome fluorescence microscope. Experiments were done in accordance to the law on animal experimentation (article 7; D.L. 116/92) under the Animal Protocol approved by the Italian Ministry of Health.

### Statistical analysis

Statistical significance was determined by a two-tailed paired Student's *t*-test. *P*-values <0.05 were considered as statistically significant. Error bars represent s.e.m.

### Data availability

Sequence data that support the findings of this study have been deposited to the GEO database with the accession code GSE79796. The authors confirm that all other data appears in the article and Supplementary Information.

## Additional information

**How to cite this article:** Fiorenzano, A. *et al*. Cripto is essential to capture mouse epiblast stem cell and human embryonic stem cell pluripotency. *Nat. Commun.* 7:12589 doi: 10.1038/ncomms12589 (2016).

## Supplementary Material

Supplementary Information Supplementary Figures 1-6, Supplementary Tables 1-2

Supplementary Data 1 List of genes differentially expressed in FBS/LIF vs F/A -induced WT cells.

Supplementary Data 2 List of genes differentially expressed in Serum/LIF vs F/A -induced Cripto KO cells.

Supplementary Data 3 List of genes differentially expressed in WT and Cripto KO F/A -treated cells.

Supplementary Data 4 Sketch of the code used for carrying out RNA-Seq statistical analysis.
