# Supplementary material for: Cripto is essential to capture mouse epiblast stem cell and human embryonic stem cell pluripotency
Source: Nat Commun. 2016 Sep 2;7:12589. doi: 10.1038/ncomms12589 (PMC5025790; doi:10.1038/ncomms12589)
Supplement: Supplementary Information — Supplementary Figures 1-6, Supplementary Tables 1-2 [file ncomms12589-s1.pdf]

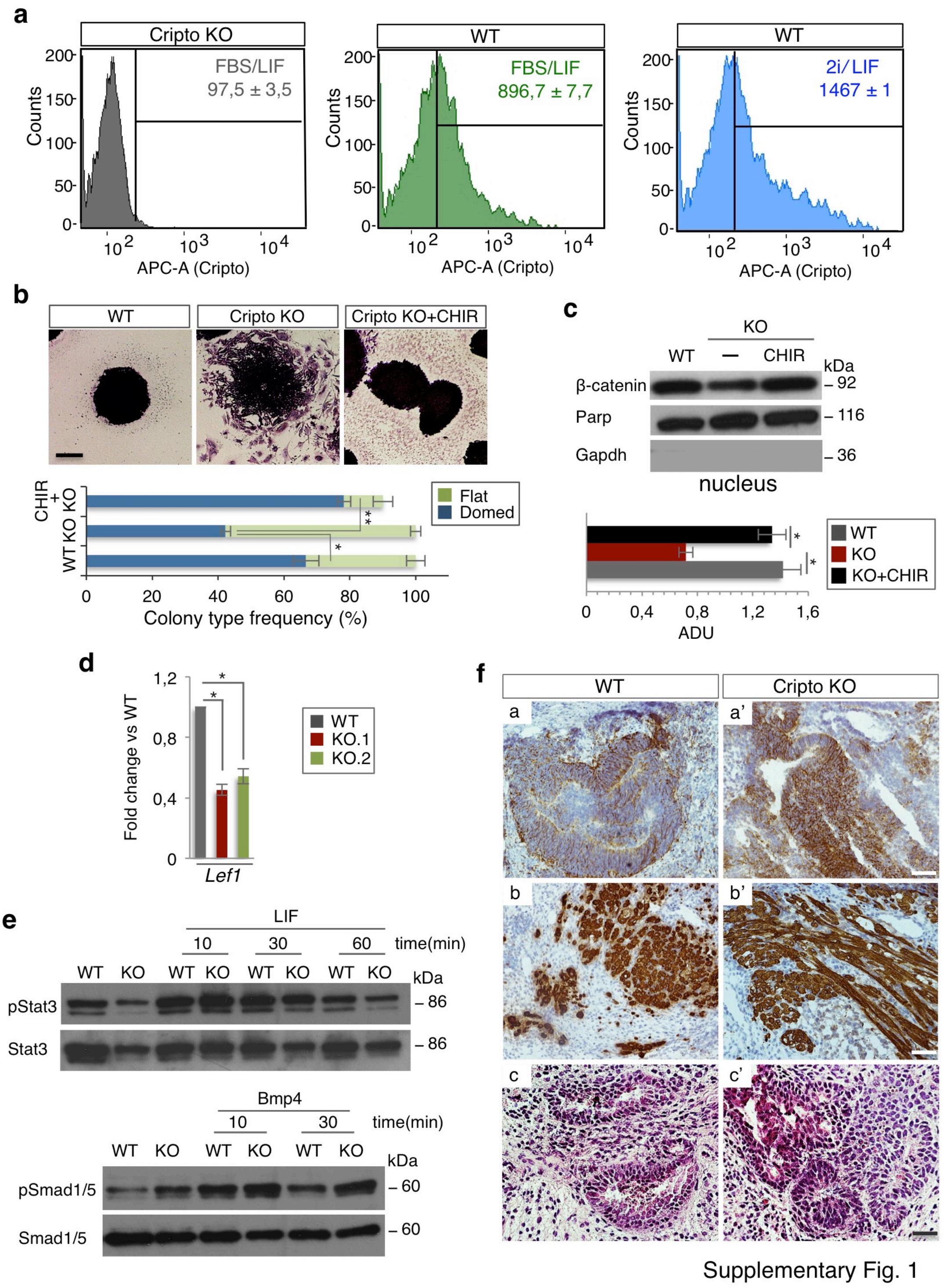

Supplementary Figure 1. Cripto regulates ESC self-renewal.

(**a**) Representative FACS plots showing Cripto expression in serum/LIF ESCs (green) and 2i LIF ESCs (blue). Cripto KO ESCs were used as negative control (dark grey). Data are shown as mean of fluorescence intensity and are expressed as mean±s.e.m. (*n*=2). (**b**) Representative pictures (scale bar, 100μm) of crystal violet-stained and colony-type frequency (domed *versus* flat) of colonies generated from WT, Cripto KO and Cripto KO+CHIR99021 (3 μM) ESCs, at day 6 after plating (~100 colonies scored/condition). Data are mean±s.e.m. (*n*=3; **P*<0.01). (**c**) Western blot analysis of nuclear β-catenin in WT, Cripto KO and Cripto KO+CHIR ESCs. Parp and Gapdh were

used as quality control of subcellular fractionation. The densitometric analysis is expressed in arbitrary unit (ADU) as β-catenin/Parp. Data are mean±s.e.m. (*n*=3; **P*<0.01). (**d**) Q-PCR of *Lef1* expression in WT ESCs and two independent Cripto KO ESC clones. Data are expressed as fold change after normalization to *Gapdh*. Data are mean±s.e.m. (*n*=3; **P*<0.01). (**e**) Western blot analysis of WT and Cripto KO ESCs stimulated with LIF and Bmp4 at the indicated time points, by using pStat3 and pSmad1/5 antibodies. Stat3 and Smad1/5 antibodies were used as loading controls. (**f**) Immunohistochemistry analysis showing tissues deriving from ectoderm (nestin; a-a'), mesoderm (MF-20; b-b') (scale bars, 75μm) and H&E staining showing endoderm derivatives (glandular epithelial structures; c-c') (scale bar, 150μm).

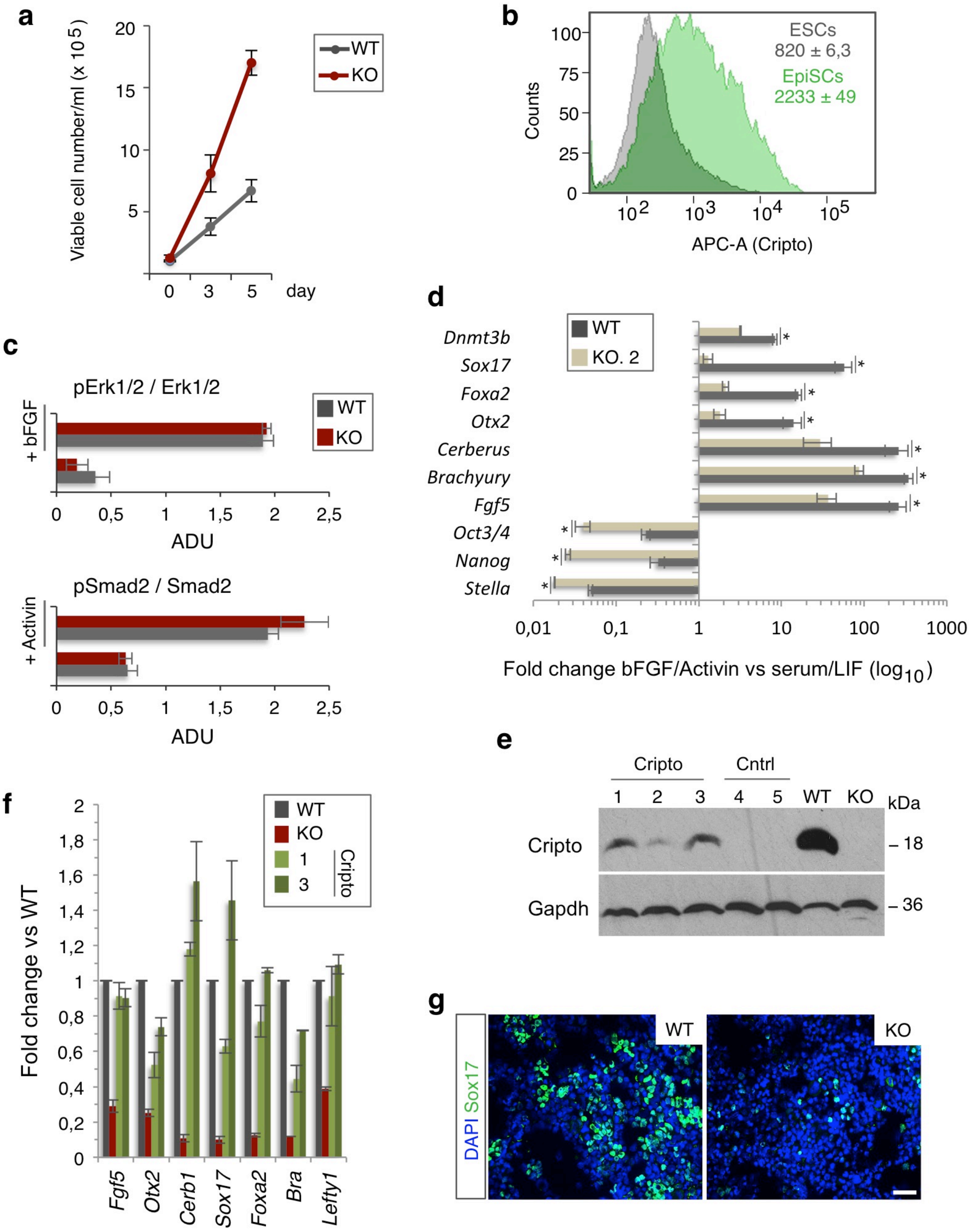

Supplementary Figure 2

Supplementary Figure 2. Cripto controls ESC→EpiSC transition

(**a**) Automated cell counting of WT and Cripto KO ESC→EpiSC transition at different time points. (**b**) Representative FACS plots showing Cripto expression in serum/LIF ESCs (grey) and F/A EpiSCs (green). Data are shown as mean of fluorescence intensity and are expressed as mean±s.e.m. (*n*=2). (**c**) Densitometric analysis of the Western Blot shown in Figure 2d expressed in arbitrary unit (ADU) as pErk1/2/Erk1/2 and pSmad2/Smad2 ratio. (**d**) Effect of F/A on the

expression of selected markers in WT and Cripto KO (clone KO.2) ESCs. Data are expressed as fold change of EpiSCs (F/A) *vs* ESCs (serum/LIF) after normalisation to *Gapdh*, and are mean±s.e.m. (n=3; *P<0.01). (**e**) Western blot analysis of Cripto protein levels in three independent Cripto KO ESC clones transfected with mouse *Cripto* cDNA expression vector [1]. WT and Cripto KO ESCs were used as positive and negative control, respectively. Gapdh was used as a loading control. (**f**) Gene expression profiles of epiblast-associated markers in F/A -induced WT, Cripto KO and *Cripto* overexpressing cells. Data are expressed as fold change compared to F/A WT EpiSCs after normalization to *Gapdh.* Data are mean±s.e.m. (n=3). (**g**) Representative immunostaining detection of Sox17 on cytospinned F/A WT and Cripto KO cells, (scale bars, 75μm). Nuclei were stained with DAPI (blue).

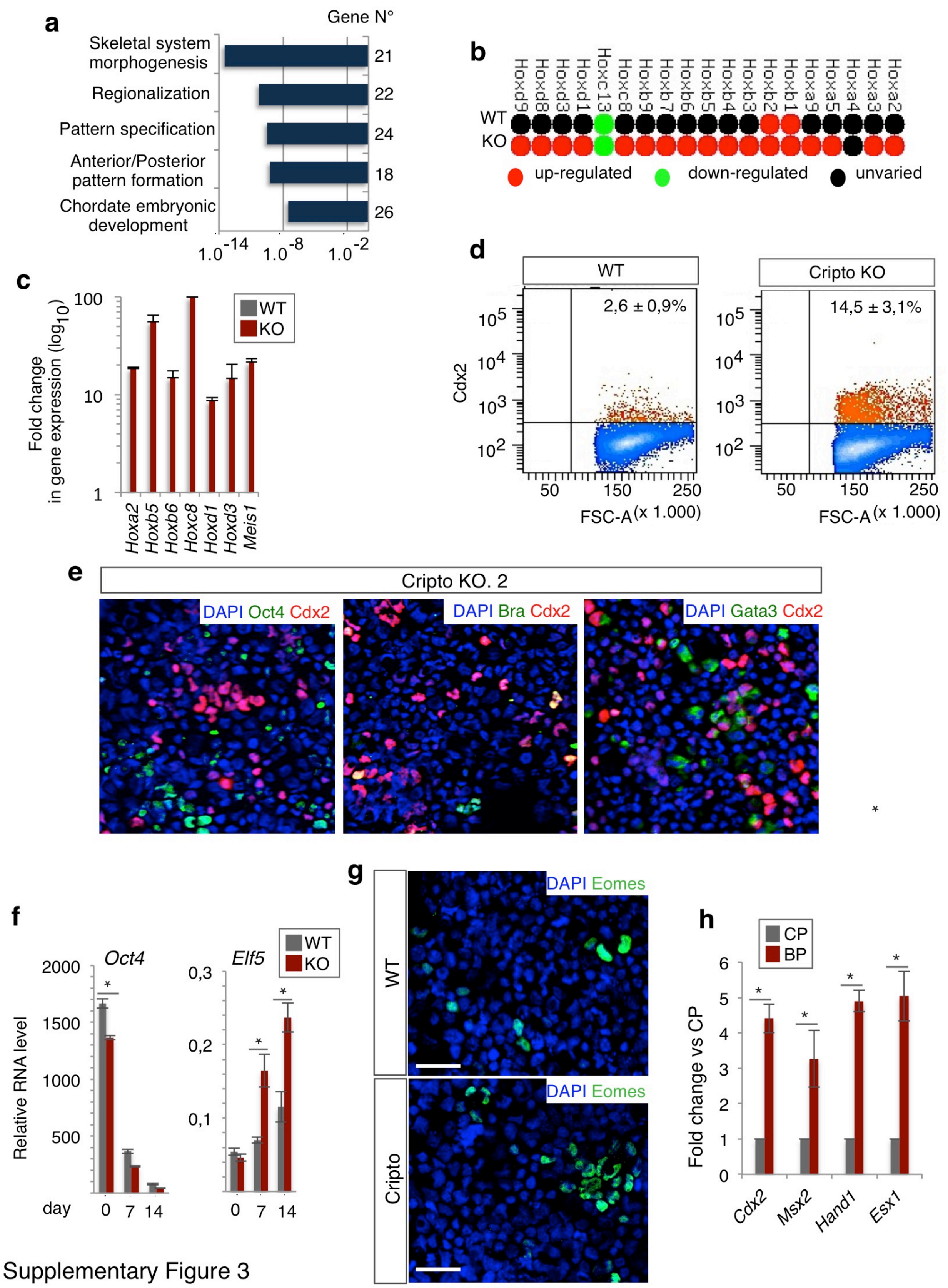

Supplementary Figure 3

Supplementary Figure 3. Cripto deficiency attenuates ESC lineage restriction

(**a**) KEGG Pathway enrichment of genes (692) uniquely deregulated in Cripto KO ESC→EpiSC transition. (**b**) Heatmap of *Hox* cluster genes in WT and Cripto KO ESC→EpiSC transition. (**c**) Q-

PCR of *Hox* genes expression in F/A Cripto KO vs WT cells. Data are expressed as fold change after normalization to *Gapdh.* (**d**) FACS -based quantification of Cdx2 positive cells in F/A WT and Cripto KO TSC cultures. Data are as mean±s.e.m. (*n*=2). (**e**) Representative pictures of Oct4/Cdx2, Bra/Cdx2 and Gata3/Cdx2 double immunostaining of F/A WT and Cripto KO (clone KO.2) cytospinned cells. Nuclei were stained with DAPI. (**f**) Q-PCR of *Oct4 and Elf5* expression at different time point during WT and Cripto KO ESC to Trophoblast Stem Cell (TSC) differentiation. Relative RNA level was normalised to *Gapdh*. Data are mean±s.e.m. (*n*=3; **P*<0.01). (**g**) Representative immunostaining detection of Eomes on cytospinned WT and Cripto KO cells (scale bars, 75μm). Nuclei were stained with DAPI. (**h**) Q-PCR of *Cdx2*, *Msx2, Hand1* and *Esx1* expression at day 6 of CP/BP-treated ESC→EpiSC transition. Data are expressed as fold change after normalization to *Gapdh.* Data are mean±s.e.m. (*n*=3; **P*<0.01).

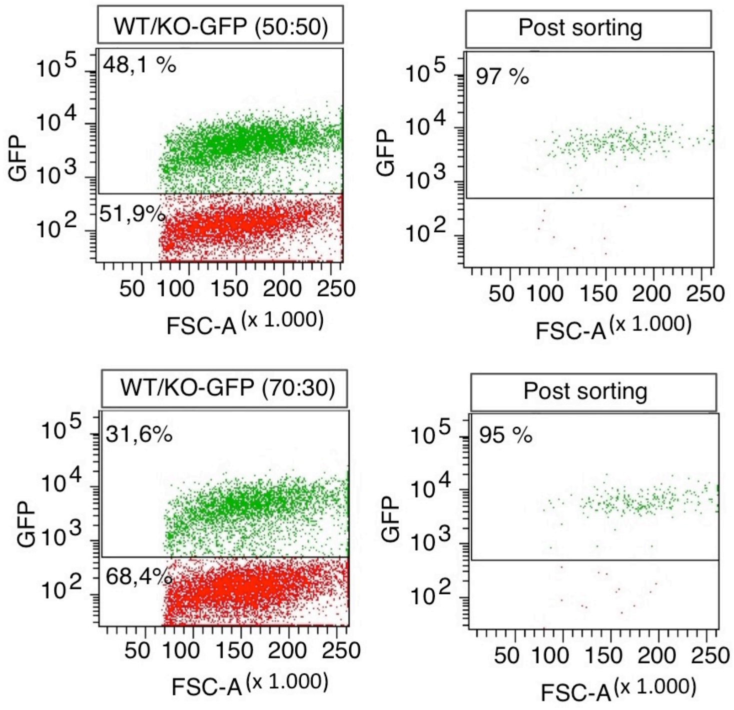

Supplementary Figure 4

Supplementary Figure 4. Non-cell autonomous contribution of Cripto in ESC→EpiSC transition

FACS-based separation of F/A WT and Cripto KO-GFP at 1:1 and 3:1 ratios and post-sorting control, respectively. Numbers are the percentage of total cells/gate.

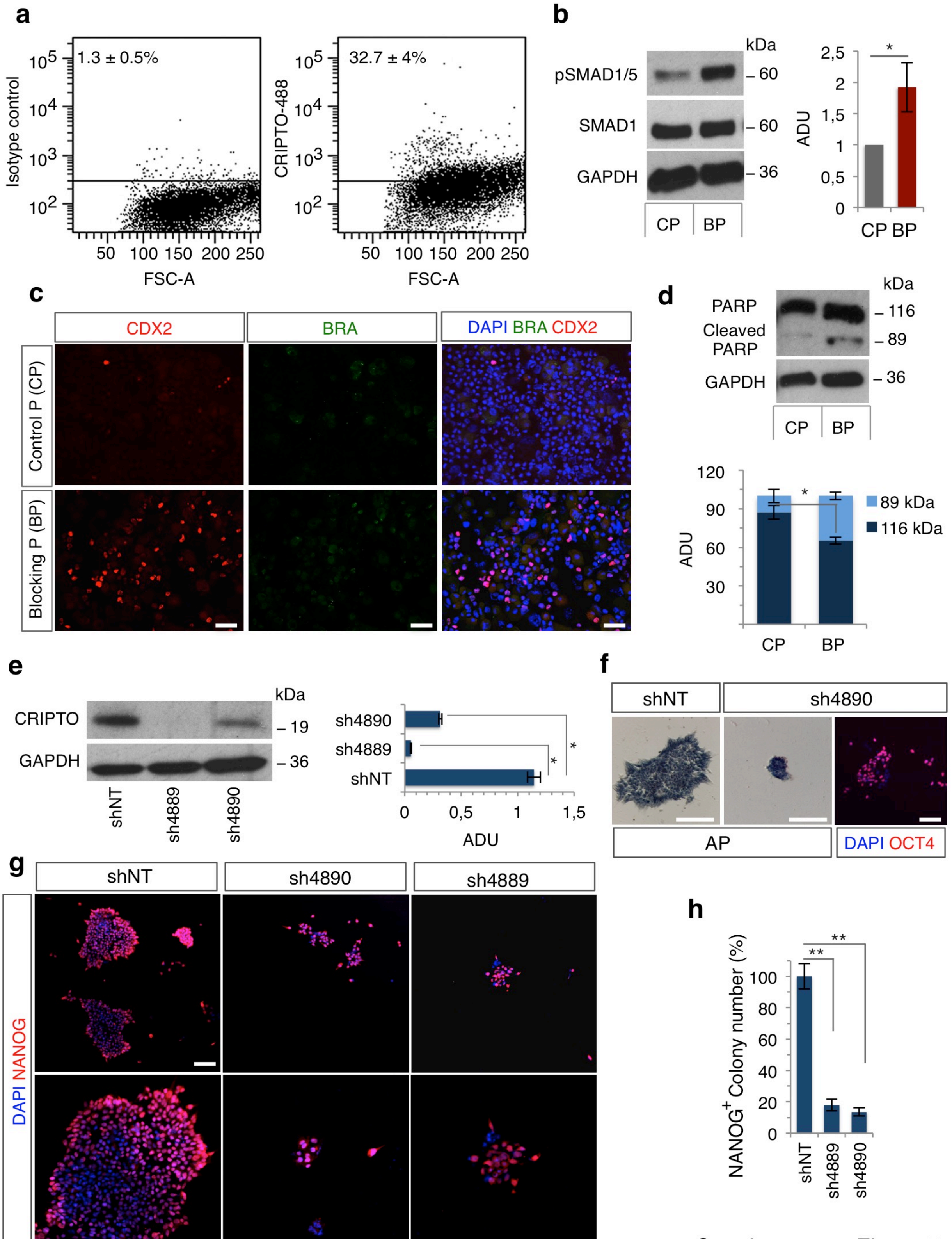

Supplementary Figure 5

Supplementary Figure 5. Cripto regulates hESCs self-renewal.

(**a**) FACS analysis of CRIPTO expression in hESCs. (**b**) Western blot analysis of pSMAD1/5 protein levels in CP- and BP- treated hESCs. SMAD1 and GAPDH antibodies were used as loading controls. The densitometric analysis is expressed in ADU as pSMAD1/5/SMAD1/5 ratio.

Data are mean±s.e.m. (*n*=2; *$P<0.01$). (**c**) Representative pictures of CDX2/BRA immunostaining in cytospinned hESCs after two passages in the presence of CP/BP (20 µM). Nuclei were stained with DAPI (blue). Scale bars, 75µm. (**d**) Western blot analysis of the full-length form (116 kDa) and caspase-specific proteolytic fragment (89 kDa) of poly (ADP-ribose) polymerase (PARP). The densitometric analysis is expressed in ADU as the PARP/GAPDH ratio. (**e**) Generation of *CRIPTO* knock down hESC lines. Western blot analysis of CRIPTO protein levels in hESCs infected with shNT (control), sh4889 or sh4890 (targeting *CRIPTO*) lentiviruses. Densitometric analysis is expressed in ADU as CRIPTO/GAPDH ratio. Data are mean±s.e.m. (*n*=2; *$P<0.01$). (**f**) Representative photomicrographs of hESC colonies derived from shNT and sh4890 lentivirus-infected hESCs, and stained for Alkaline Phosphatase (AP) and/or DAPI. Nuclei are stained with DAPI. Scale bar, 200µm. (**g**) Representative immunofluorescence photomicrographs of hESC colonies derived from shNT Control, sh4890 and sh4889 *CRIPTO* KD hESCs stained for NANOG. Scale bar, 100µm. (**h**) Quantification of NANOG+ colonies shown as percentage over shNT hESCs. Data are mean±s.e.m. (*n*=3; **$P<0.005$).

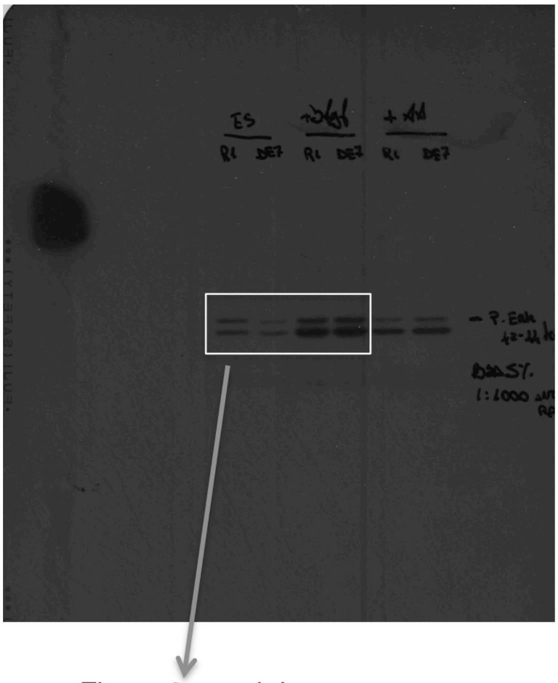

Figure 3 panel d
(pErk1/2)

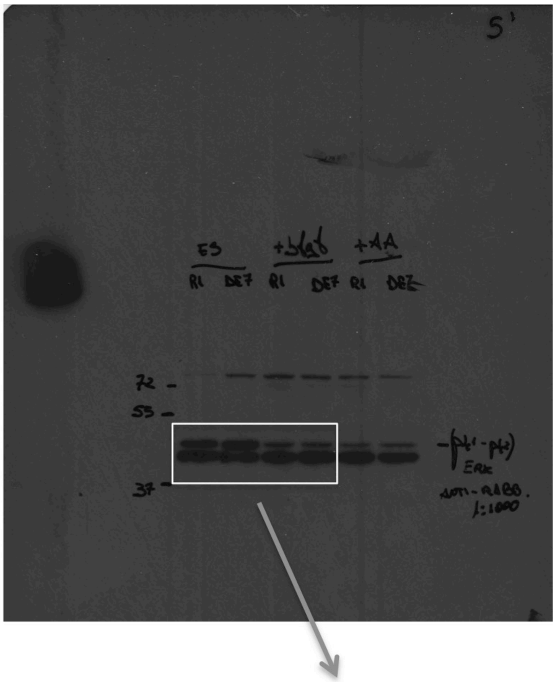

Figure 3 panel d
(Erk1/2)

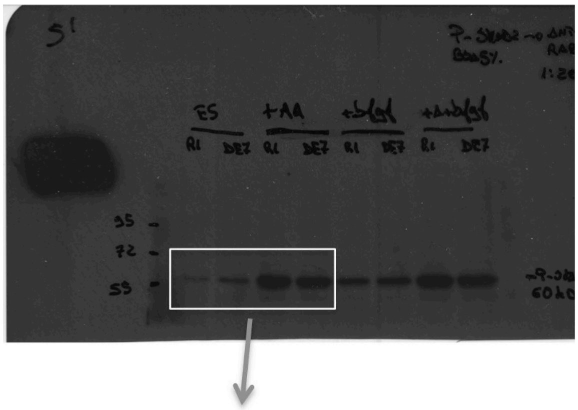

Figure 3 panel d
(pSmad2)

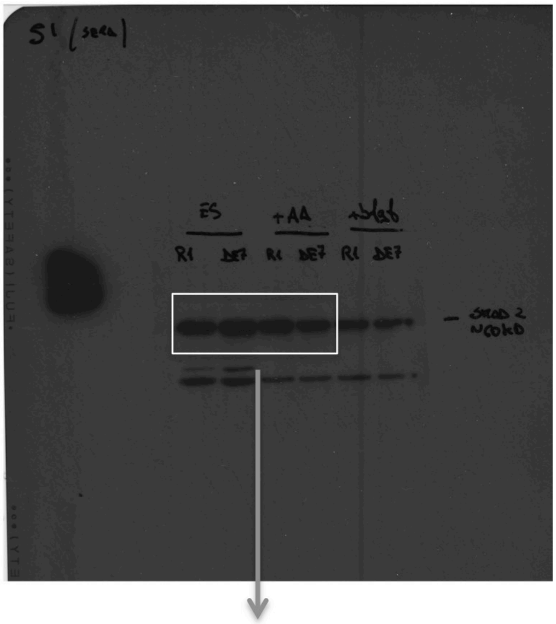

Figure 3 panel d
(Smad2)

Figure 3

Supplementary Figure 6. Full blots for data shown in the main Figure 3

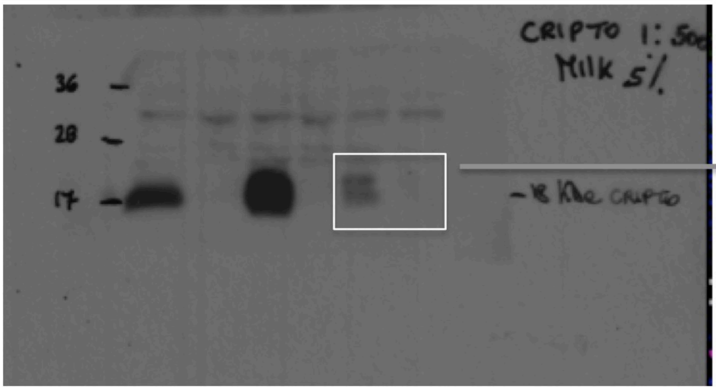

Figure 4 panel j
(Cripto)

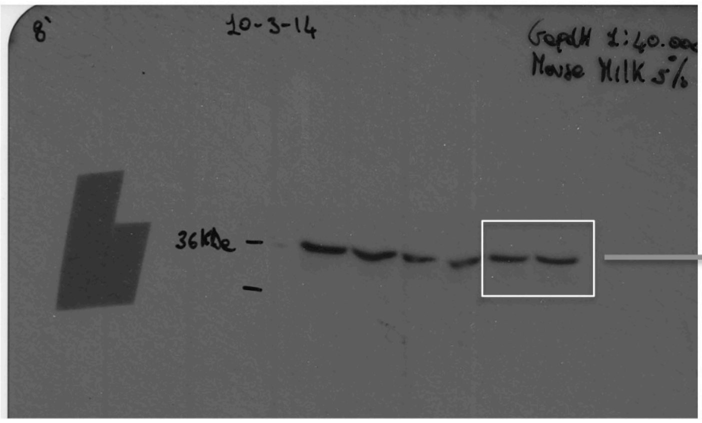

Figure 4 panel j
(Gapdh)

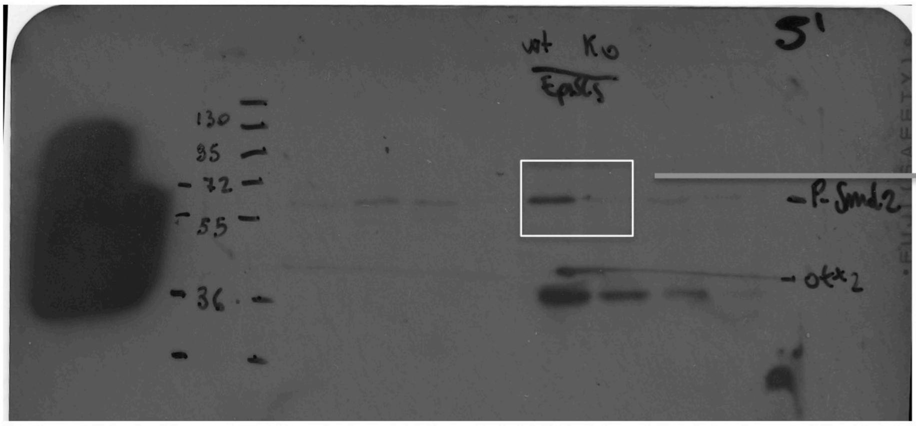

Figure 4 panel j
(pSmad2)

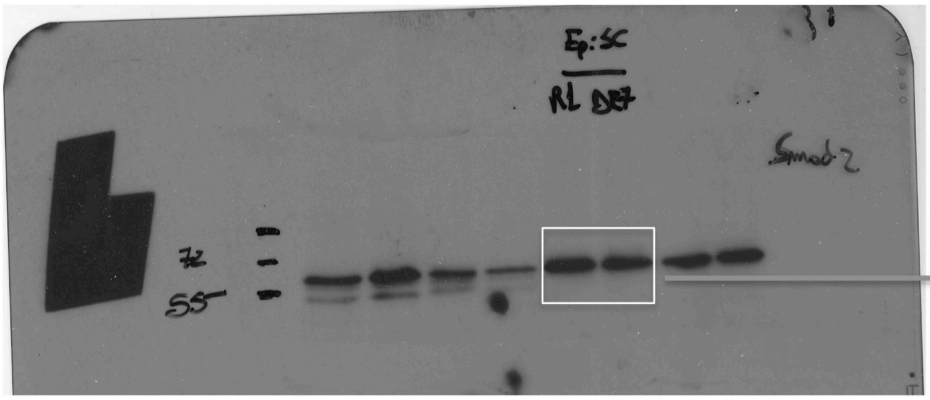

Figure 4 panel j
(Smad2)

Figure 4

Supplementary Figure 6 (continued). Full blots for data shown in the main Figure 4

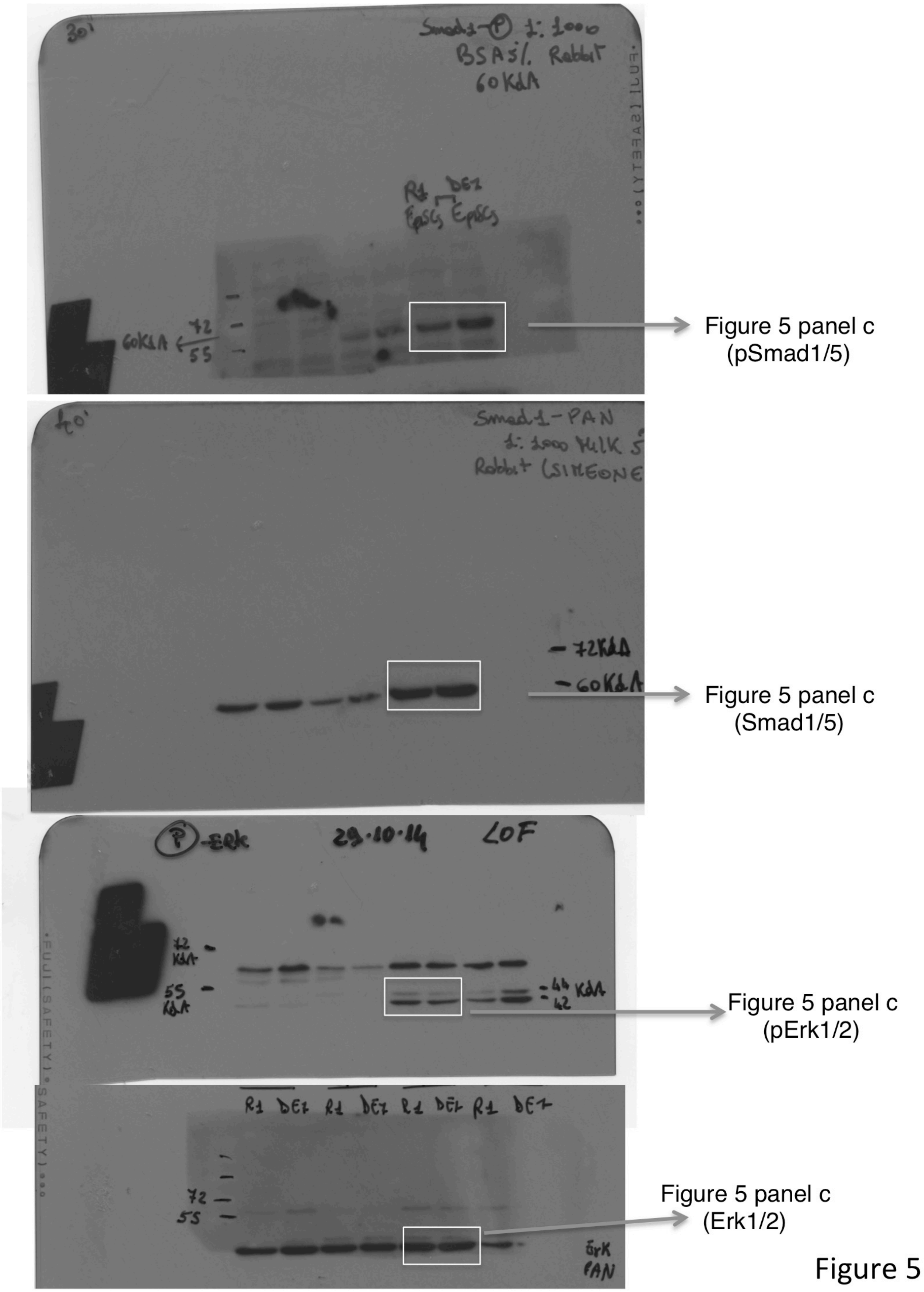

Supplementary Figure 6 (continued). Full blots for data shown in the main Figure 5

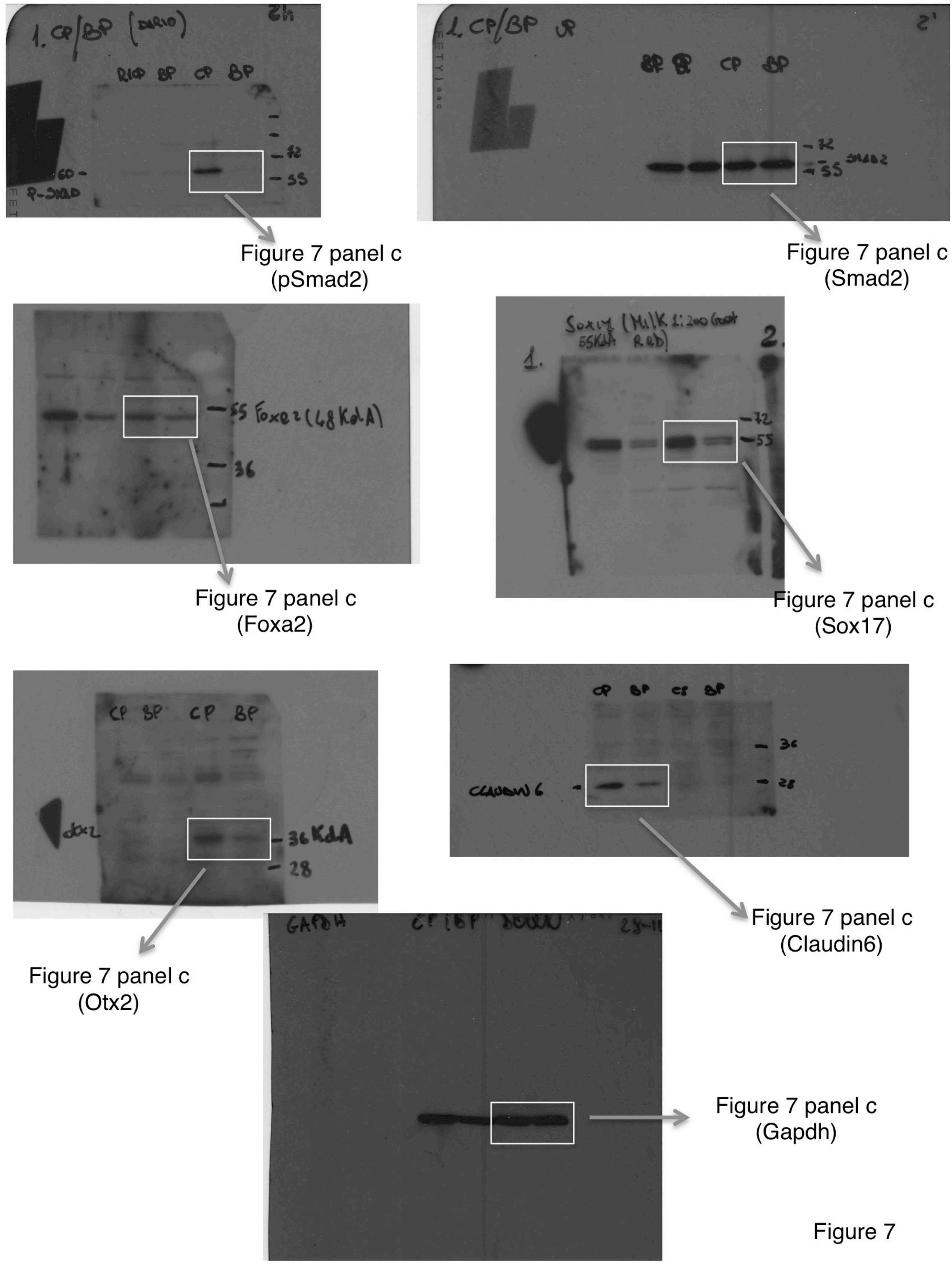

Supplementary Figure 6 (continued). Full blots for data shown in the main Figure 7

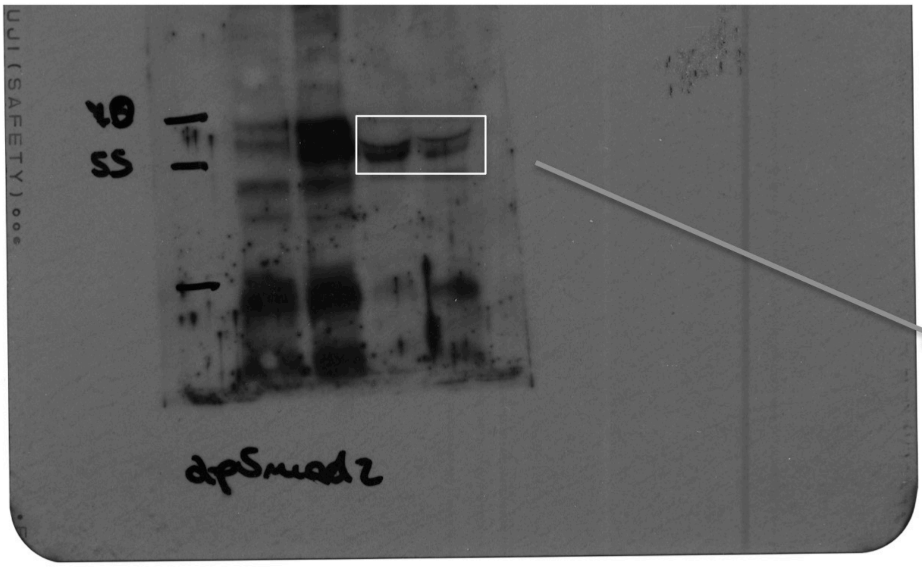

Figure 8 panel e
(pSmad2)

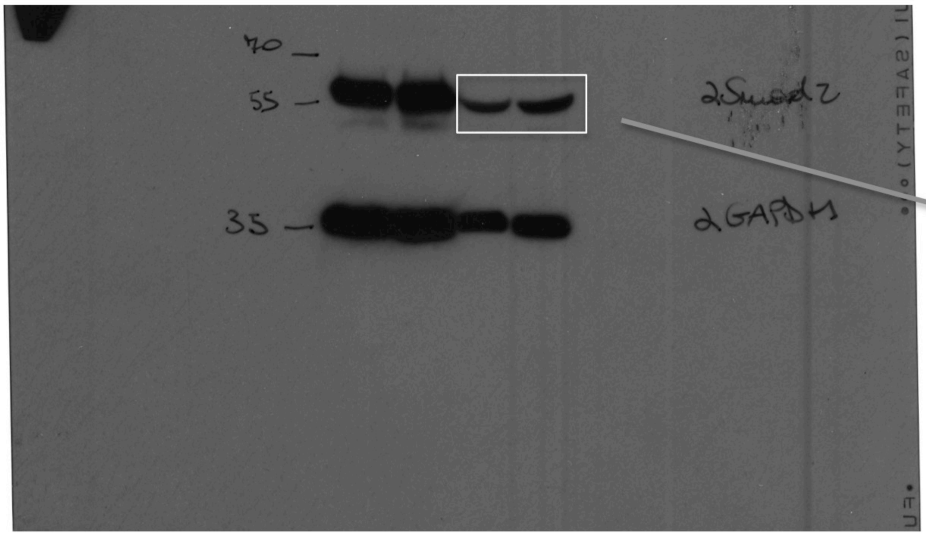

Figure 8 panel e
(Smad2)

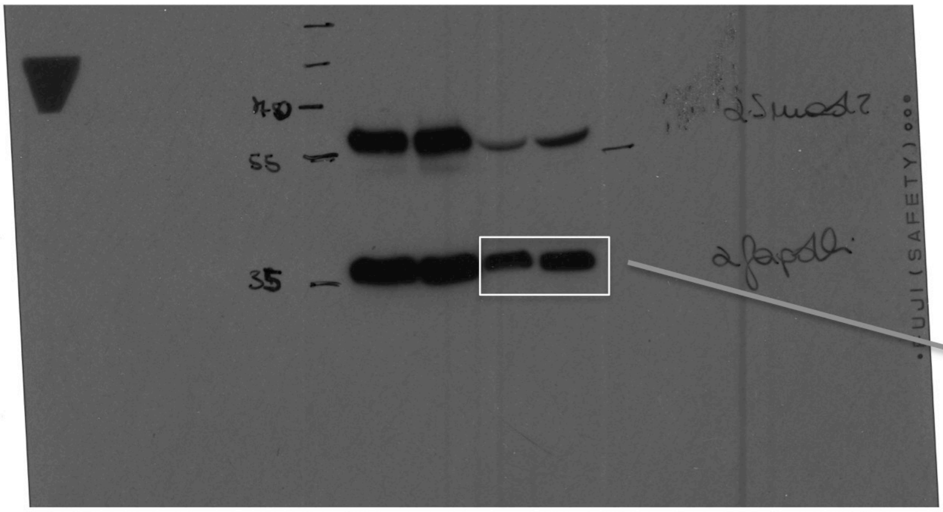

Figure 8 panel e
(Gapdh)

Figure 8

Supplementary Figure 6 (continued). Full blots for data shown in the main Figure 8

Supplementary Table 1

List of antibodies used throughout the study

ANTIBODY	SOURCE	CAT. NO.	APPLICATION
**mCripto -APC**	R&D	FAB1538A	FACS (10µl/1x10^6cells)
**mCripto**	Home Made		IF (1 µg ml^{-1})
**mCripto**	R&D	AF1538	WB (1 µg ml^{-1})
**hCripto -488**	R&D	AF1538	IF (1 µg ml^{-1})
**hCripto**	R&D	MAB2772	WB (1 µg ml^{-1})
**Phospho-Stat3**	Cell Signaling	9145	WB (1:2000)
**Stat3**	Cell Signaling	3108	WB (1:2000)
**Phospho-Smad1/5**	Cell Signaling	9516	WB (1:1000)
**Smad1**	Cell Signaling	9743	WB (1:1000)
**Phospho-Erk1/2**	Cell Signaling	9101	WB (1:1000)
**Erk1/2**	Cell Signaling	9102	WB (1:1000)
**Phospho-Smad2**	Cell Signaling	3108	WB (1:200)
**Smad2**	Cell Signaling	3103	WB (1:1000)
**Nanog**	Cell Signaling	8822	IF (1:400)
**hNANOG**	R&D	AF1997	IF on hESCs (1:20)
**Nanog**	eBioscience	14-5761	IF on mouse embryos
**Oct4**	Santa Cruz B.T.	sc-8628	IF (1:400 on mESCs; 1:100 on hESCs)
**Brachyury**	Santa Cruz B.T.	sc-17745	IF (1:200)
**Otx2**	R&D	AF1979	IF (1:200) WB (1:500)
**Claudin6**	Santa Cruz B.T.	sc-17669	IF (1:200) WB (1:500)
**Foxa2**	abcam	ab40874	IF (1:200) WB (1:500)
**Sox17**	Santa Cruz B.T.	sc-17318	IF (1:200) WB (1:500)
**Cdx2**	abcam	ab86949	IF, FACS
**Gata3**	Santa Cruz B.T.	sc-268	IF (1:200)
**Mf20**	Hybridoma Bank		IHC (1:50)
**Nestin**	Santa Cruz B.T.	sc-33677	IHC (1:400)
**PARP**	Cell Signaling	9542	WB (1:1000)
**Pecam 1**	R&D	AF3628	IF
**Dab2**	BD Biosciences	610464	IF
**βCatenin**	BD Biosciences	610153	WB(1.2000)

**GFP**	abcam	ab6673	IF (1:1000)
**PGC-1b**	abcam	ab176328	IF (1:500)

Supplementary Table 2

Sequences of qPCR primers

GENE NAME	FORWARD PRIMER	REVERSE PRIMER
Cripto	TGTTCGCAAAGAGCACTGTGG	TGAGGTCCTGGTCCATCACTTGAC
Gapdh	TGGGGGAACTTAAAGTGCAG	GATGTAGGCAGCTGTCATTC
Stella	TTCCGAGCTAGCTTTTGAGG	ACACCGGGGTTTAGGGTTAG
Nanog	AAGTACCTCAGCCTCCAGCA	GTGCTGAGCCCTTCTGAATC
Esrrb	GGCCACCAATGAATGTGAG	AGCCGTCGCTTGTACTTCTG
Oct3/4	TCAGCTTGGGCTAGAGAAGG	TGACGGGAACAGAGGGAAAG
Lef1	GCAGCTATCAACCAGATCC	GATGTAGGCAGCTGTCATTC
Brachyury	GAACCTCGGATTCACATCGT	TTCTTTGGCATCAAGGAAGG
Fgf5	CAAAGTCAATGGCTCCCACGAAG	CTACAATCCCCTGAGACACAGCA
Otx2	GGTATTGGACTTGCTGCATCC	CGAGCTGTGCCCTGATAAATG
Lefty1	AGCTCAAGGCAATTGTGACC	TCATCTCTGAGGCGACACAC
Cer1	AGGAGGAAGCCAAGAGGTTC	CATTTGCCAAAGCAAAAGGTT
Sox17	AGCTAAGCAAGATGCTAGGCAAG	TCTCTGCCAAGGTCAACGC
Dnmt3b	GCCAGCCTCACGACAGGAAAC	GACTGGGGGTGAGGGAGCATC
Foxa2	ACCTGAGTCCGAGTCTGAGC	TGTAGCTGCGTCGGTATGTC
Cdx2	AGGAAGCCAAGGTGAAAACCA	CAGCCAGCTCACTTTTCCTC
Gata3	GGGCTACGGTGCAGAGGTAT	TGGATGGACGTCTTGGAGAA
Msx2	CCTCGGTCAAGTCGGAAAAT	GGTACTGTTTCTGGCGGAAC
Hand1	GGATGCACAAGCAGGTGAC	GCGCCCTTTAATCCTCTTCT
Hoxa2	TCCCTGGATGAAGGAGAAGA	GGTGTACGCGGTTCTCAGAC
Hoxb5	AGGGGCAGACTCCACAGATA	GGGTCAGGTAGCGATTGAAG
Hoxb6	AAGAGCGTGTTCGGAGAGAC	CAGGGTCTGGTAGCGTGTGT
Hoxc8	GTCTCCCAGCCTCATGTTTC	GGGCGTGAGAGACTTCAATC
Hoxd1	CAGCACTTTCGAGTGGATGA	GCTCTGTCAGTTGCTTGGTG
Hoxd3	CAAACAGATCTTCCCCTGGA	ACCAGCTGAGCACTCGTGTA
Meis1	GCCAATGGGAGGTTTTGTAA	GCATCTGGGCTTGGGTATAA
Pgcb	GTGCCAGGTGCTGACGAGAA	AGTGTATCTGGGCCAACGGAA
Cox7a1	GTGGCAGAGAAGCAGAAGCTC	CCAGCCCAAGCAGTATAAGCAGT
Eomes	GGCAAAGCGGACAATAACAT	AGCCTCGGTTGGTATTTGTG
Tead4	GCACCATTACCTCCAACGAG	GATCAGCTCATTCCGACCAT

Elf5	GGACCGATCTGTTCAGCAAT	CTTGTACTGGTCGCAGCAGA
