# Supplementary material for: Cripto is essential to capture mouse epiblast stem cell and human embryonic stem cell pluripotency
Source: Nat Commun. 2016 Sep 2;7:12589. doi: 10.1038/ncomms12589 (PMC5025790; doi:10.1038/ncomms12589)
Supplement: Supplementary Data 4 — Sketch of the code used for carrying out RNA-Seq statistical analysis. [file ncomms12589-s5.html]

The project report of "Cripto is essential to capture mouse epiblast stem cell and human embryonic stem cell pluripotency"


## Supplementary file containing the sketches of the R code used in "Cripto is essential to capture mouse epiblast stem cell and human embryonic stem cell pluripotency"

### This report describes some main sketches of the R code used for the RNA-seq gene expression analysis. The analysis starts from the raw count file obtained by HTseq (version 0.6.1p1) and was performed using RNASeqGUI (version 0.99.3) inside an R console (version 3.1.0).

- The first step regards the usage of the Full Quantile Normalization procedure provided by the function "normalize.quantiles" of the "preprocessCore" package (version 1.28.0). To such purpose, in the Data Exploration Interface of RNASeqGUI, we loaded the count table, "T\_counts.txt", as input file, and we clicked the FQUA button. The normalized counts were saved in the "T\_counts.txt\_FQUA.txt" file.

We loaded the following count file: T\_counts.txt
.
This R code has been run:

```
  require(preprocessCore)
  x = read.table('T_counts.txt',header=TRUE,row.names=1)
  x=data.matrix(x, rownames.force = NA)
  the.file ='T_counts.txt'
  Project ='min'
  myfqua = normalize.quantiles(x,copy=TRUE)
```

- The second step consists in the usage of the Proportion test ("type3 <- TRUE" in the code below) as filtering procedure provided by the function "filtered.data" of the "NOISeq" package (version 2.8.0). Therefore, in the Filtering Interface, we loaded the normalized counts file (T\_counts.txt\_FQUA.txt) and we clicked the Filter button. Results were saved in the "Proportion\_T\_counts.txt\_FQUA.txt".

We loaded the following file: `/RNASeqGUI_Projects/min/Results/T_counts.txt_FQUA.txt`. We used the following settings:

conditions: `WT_F/A, WT_Serum/LIF, Cripto_K_F/A, Cripto_KO_Serum/LIF`
, type1:`FALSE`
, type2:`FALSE`
, type3:`TRUE`
, norm: `TRUE`
, Project:`min`
, cpm:`1`

This R code has been run:

```
 require(NOISeq)
 the.file='T_counts.txt_FQUA'
 x = read.table('/RNASeqGUI_Projects/min/Results/T_counts.txt_FQUA.txt',header=TRUE,row.names=1)
 the.file ='/RNASeqGUI_Projects/min/Results/T_counts.txt_FQUA.txt'
 Project ='min'
 conditions <- c('WT_F/A', 'WT_Serum/LIF', 'Cripto_K_F/A', 'Cripto_KO_Serum/LIF')
 type1 <- FALSE
 type2 <- FALSE
 type3 <- TRUE
 norm <- TRUE
 cpm <- 1
 depth = NULL
 for( i in 1:ncol(x) ){depth[i] = sum(x[,i])}
 filtered_x = filtered.data(x, factor = conditions, norm = norm, depth = depth, method = 3, cpm = cpm)
```

- The third step regards the analysis of differentially expressed genes. We used full the quantile filtered normalized counts (file Proportion\_T\_counts.txt\_FQUA.txt) to perform the analysis. We carried out four different comparisons, such as:

  WT\_F/A vs WT\_Serum/LIF,

  WT\_Serum/LIF vs Cripto\_KO\_Serum/LIF,

  Cripto\_KO\_F/A vs Cripto\_KO\_Serum/LIF,

  WT\_F/A vs Cripto\_KO\_F/A.

  For simplicity, we created four different count files from the Proportion\_T\_counts.txt\_FQUA.txt, such as:

  WT\_F/A\_WT\_Serum/LIF.txt,

  WT\_Serum/LIF\_Cripto\_KO\_Serum/LIF.txt,

  Cripto\_KO\_F/A\_Cripto\_KO\_Serum/LIF.txt,

  WT\_F/A\_Cripto\_KO\_F/A.txt,

  Each file above contains the samples to compare.

We opened the *Data Analysis Interface* of RNASeqGUI and then we opened the *NOISeq Interface*. For the first comparison, we loaded the following count file: `WT_F/A_WT_Serum/LIF.txt`. We used the following settings:
 prob: `0.95`, Project: `min`,
Tissue:  `WT_F/A, WT_Serum/LIF`,
TissueRun:  `1, 1`. 

This R code has been run:

```
 require(NOISeq)
 x = read.table('WT_F/A_WT_Serum/LIF.txt',header=TRUE,row.names=1)
 the.file ='WT_F/A_WT_Serum/LIF.txt'
 Project ='min'
 replicate_type ='technical'
 p='0.95'
 conditions= c('WT_F/A','WT_Serum/LIF') 
 TissueRuns= c('1','1')  
  p = as.numeric(p)
  myfactors = data.frame(Tissue = conditions, TissueRun = TissueRuns)
  mydata <- NOISeq::readData(data=x, factors=myfactors)
  mynoiseq = noiseq(mydata, k = 0.5, norm = 'rpkm', factor='Tissue', pnr = 0.2, nss = 5, v = 0.02, lc = 1, 
  replicates = replicate_type)
  list_DE_NOISEQ = subset(mynoiseq@results[[1]], prob > p) # select significant genes
```

- For the second comparison, we loaded the following count file: `WT_Serum/LIF_Cripto_KO_Serum/LIF.txt`. In the *NOISeq Interface*, we clicked the **Run NOISeq** button. We used the following settings:

prob: `0.95`, Project: `min`,
Tissue: `WT_Serum/LIF, Cripto_KO_Serum/LIF`,
TissueRun: `1, 1`.

This R code has been run:

```
 require(NOISeq)
 x = read.table('WT_Serum/LIF_Cripto_KO_Serum/LIF.txt',header=TRUE,row.names=1)
 the.file ='WT_Serum/LIF_Cripto_KO_Serum/LIF.txt'
 Project ='min'
 replicate_type ='technical'
 p='0.95'
 conditions= c('WT_Serum/LIF','Cripto_KO_Serum/LIF') 
 TissueRuns= c('1','1') 
  p = as.numeric(p)
  myfactors = data.frame(Tissue = conditions, TissueRun = TissueRuns)
  mydata <- NOISeq::readData(data=x, factors=myfactors)
  mynoiseq = noiseq(mydata, k = 0.5, norm = 'rpkm', factor='Tissue', pnr = 0.2, nss = 5, v = 0.02, lc = 1,
  replicates = replicate_type)
  list_DE_NOISEQ = subset(mynoiseq@results[[1]], prob > p) # select significant genes
```

- For the third comparison, we loaded the following count file:  `Cripto_KO_F/A_Cripto_KO_Serum/LIF.txt`. In the *NOISeq Interface*, we clicked the **Run NOISeq** button. We used the following settings:

prob: `0.95`, Project: `min`,
Tissue:  `Cripto_KO_F/A, Cripto_KO_Serum/LIF`,
TissueRun:  `1, 1`.

This R code has been run:

```
 require(NOISeq)
 x = read.table('Cripto_KO_F/A_Cripto_KO_Serum/LIF.txt',header=TRUE,row.names=1)
 the.file ='Cripto_KO_F/A_Cripto_KO_Serum/LIF.txt'
 Project ='min'
 replicate_type ='technical'
 p='0.95'
 conditions= c('Cripto_KO_F/A','Cripto_KO_Serum/LIF') 
 TissueRuns= c('1','1') 
  p = as.numeric(p)
  myfactors = data.frame(Tissue = conditions, TissueRun = TissueRuns)
  mydata <- NOISeq::readData(data=x, factors=myfactors)
  mynoiseq = noiseq(mydata, k = 0.5, norm = 'rpkm', factor='Tissue', pnr = 0.2, nss = 5, v = 0.02, lc = 1,
  replicates = replicate_type)
  list_DE_NOISEQ = subset(mynoiseq@results[[1]], prob > p) # select significant genes
```

- For the fourth comparison, we loaded the following count file:  `WT_F/A_Cripto_KO_F/A.txt`. In the *NOISeq Interface*, we clicked the **Run NOISeq** button. We used the following settings:

prob: `0.95`, Project: `min`,
Tissue: `WT_F/A,Cripto_KO_F/A`,
TissueRun: `1 ,1`.

This R code has been run:

```
 require(NOISeq)
 x = read.table('WT_F/A_Cripto_KO_F/A.txt',header=TRUE,row.names=1)
 the.file ='WT_F/A_Cripto_KO_F/A.txt'
 Project ='min'
 replicate_type ='technical'
 p='0.95'
 conditions= c('WT_F/A','Cripto_KO_F/A') 
 TissueRuns= c('1','1') 
  p = as.numeric(p)
  myfactors = data.frame(Tissue = conditions, TissueRun = TissueRuns)
  mydata <- NOISeq::readData(data=x, factors=myfactors)
  mynoiseq = noiseq(mydata, k = 0.5, norm = 'rpkm', factor='Tissue', pnr = 0.2, nss = 5, v = 0.02, lc = 1, 
  replicates = replicate_type)
  list_DE_NOISEQ = subset(mynoiseq@results[[1]], prob > p) # select significant genes
```

Finally, since the output of NOISeq is not deterministic, for each comparison we run NOISeq ten times. Each time, we set a different seed. The seeds used are the following ones: the default value of R version 3.1.0, 10, 21, 33, 44, 55, 66, 77, 88, 99. Then, for each gene we calculated the mean of the ten posterior probabilities obtained so far. We considered a gene as differentially expressed across the samples if the mean of the ten posterior probabilities is greater or equal to 0.95.

All package versions loaded by RNASeqGUI at the time of the analysis are reported below.

```
R version 3.1.0 (2014-04-10)
Platform: x86_64-unknown-linux-gnu (64-bit)

locale:
 [1] LC_CTYPE=en_US.UTF-8       LC_NUMERIC=C              
 [3] LC_TIME=en_US.UTF-8        LC_COLLATE=en_US.UTF-8    
 [5] LC_MONETARY=en_US.UTF-8    LC_MESSAGES=en_US.UTF-8   
 [7] LC_PAPER=en_US.UTF-8       LC_NAME=C                 
 [9] LC_ADDRESS=C               LC_TELEPHONE=C            
[11] LC_MEASUREMENT=en_US.UTF-8 LC_IDENTIFICATION=C       

attached base packages:
[1] splines   parallel  stats     graphics  grDevices utils     datasets 
[8] methods   base     

other attached packages:
 [1] car_2.0-21                NOISeq_2.8.0             
 [3] ineq_0.2-13               e1071_1.6-4              
 [5] ReportingTools_2.4.0      RSQLite_0.11.4           
 [7] DBI_0.3.1                 knitr_1.6                
 [9] Rsubread_1.14.2           digest_0.6.4             
[11] scatterplot3d_0.3-35      preprocessCore_1.28.0    
[13] leeBamViews_1.0.0         EDASeq_1.10.0            
[15] aroma.light_2.0.0         matrixStats_0.10.0       
[17] ShortRead_1.22.0          GenomicAlignments_1.0.6  
[19] BSgenome_1.32.0           RColorBrewer_1.0-5       
[21] gplots_2.14.2             pasilla_0.4.0            
[23] DEXSeq_1.10.8             BiocParallel_0.6.1       
[25] DESeq2_1.4.5              RcppArmadillo_0.4.450.1.0
[27] Rcpp_0.11.3               Rsamtools_1.16.1         
[29] Biostrings_2.32.1         XVector_0.4.0            
[31] GenomicFeatures_1.16.3    AnnotationDbi_1.26.1     
[33] Biobase_2.24.0            GenomicRanges_1.16.4     
[35] GenomeInfoDb_1.0.2        IRanges_1.22.10          
[37] BiocGenerics_0.10.0       RGtk2_2.20.31            
[39] RNASeqGUI_0.99.3         

loaded via a namespace (and not attached):
 [1] acepack_1.3-3.3          annotate_1.42.1          AnnotationForge_1.6.1   
 [4] base64enc_0.1-2          BatchJobs_1.4            BBmisc_1.7              
 [7] biomaRt_2.20.0           biovizBase_1.12.3        bitops_1.0-6            
[10] brew_1.0-6               Category_2.30.0          caTools_1.17.1          
[13] checkmate_1.4            class_7.3-10             cluster_1.15.2          
[16] codetools_0.2-8          colorspace_1.2-4         DESeq_1.16.0            
[19] dichromat_2.0-0          edgeR_3.6.8              evaluate_0.5.5          
[22] fail_1.2                 foreach_1.4.2            foreign_0.8-61          
[25] formatR_1.0              Formula_1.1-2            gdata_2.13.3            
[28] genefilter_1.46.1        geneplotter_1.42.0       ggbio_1.12.10           
[31] ggplot2_1.0.0            GO.db_2.14.0             GOstats_2.30.0          
[34] graph_1.42.0             grid_3.1.0               gridExtra_0.9.1         
[37] GSEABase_1.26.0          gtable_0.1.2             gtools_3.4.1            
[40] Hmisc_3.14-5             hwriter_1.3.2            iterators_1.0.7         
[43] KernSmooth_2.23-12       lattice_0.20-29          latticeExtra_0.6-26     
[46] limma_3.20.9             locfit_1.5-9.1           markdown_0.7.4          
[49] MASS_7.3-35              Matrix_1.1-4             munsell_0.4.2           
[52] nnet_7.3-8               PFAM.db_2.14.0           plyr_1.8.1              
[55] proto_0.3-10             RBGL_1.40.1              RCurl_1.95-4.3          
[58] reshape2_1.4             R.methodsS3_1.6.1        R.oo_1.18.0             
[61] rpart_4.1-8              rtracklayer_1.24.2       R.utils_1.33.0          
[64] scales_0.2.4             sendmailR_1.2-1          statmod_1.4.20          
[67] stats4_3.1.0             stringr_0.6.2            survival_2.37-7         
[70] tools_3.1.0              VariantAnnotation_1.10.5 XML_3.98-1.1            
[73] xtable_1.7-4             zlibbioc_1.10.0
```
